# The TrkAIII Oncoprotein Inhibits Mitochondrial Free Radical ROS-Induced Death of SH-SY5Y Neuroblastoma Cells by Augmenting SOD2 Expression and Activity at the Mitochondria, within the Context of a Tumour Stem Cell-like Phenotype

**DOI:** 10.1371/journal.pone.0094568

**Published:** 2014-04-15

**Authors:** Pierdomenico Ruggeri, Antonietta R. Farina, Natalia Di Ianni, Lucia Cappabianca, Marzia Ragone, Giulia Ianni, Alberto Gulino, Andrew R. Mackay

**Affiliations:** 1 Department of Applied Clinical and Biotechnological Sciences, University of L’Aquila, L’Aquila, Italy; 2 Department of Medical-Surgical Science and Biotechnology, University of Rome “La Sapienza”, Latina, Italy; 3 Department of Experimental Medicine, University of Rome “La Sapienza”, Rome, Italy; North Carolina State University, United States of America

## Abstract

The developmental and stress-regulated alternative TrkAIII splice variant of the NGF receptor TrkA is expressed by advanced stage human neuroblastomas (NBs), correlates with worse outcome in high TrkA expressing unfavourable tumours and exhibits oncogenic activity in NB models. In the present study, we report that constitutive TrkAIII expression in human SH-SY5Y NB cells inhibits Rotenone, Paraquat and LY83583-induced mitochondrial free radical reactive oxygen species (ROS)-mediated death by stimulating SOD2 expression, increasing mitochondrial SOD2 activity and attenuating mitochondrial free radical ROS production, in association with increased mitochondrial capacity to produce H_2_O_2_, within the context of a more tumour stem cell-like phenotype. This effect can be reversed by the specific TrkA tyrosine kinase inhibitor GW441756, by the multi-kinase TrkA inhibitors K252a, CEP-701 and Gö6976, which inhibit SOD2 expression, and by siRNA knockdown of SOD2 expression, which restores the sensitivity of TrkAIII expressing SH-SY5Y cells to Rotenone, Paraquat and LY83583-induced mitochondrial free radical ROS production and ROS-mediated death. The data implicate the novel TrkAIII/SOD2 axis in promoting NB resistance to mitochondrial free radical-mediated death and staminality, and suggest that the combined use of TrkAIII and/or SOD2 inhibitors together with agents that induce mitochondrial free radical ROS-mediated death could provide a therapeutic advantage that may also target the stem cell niche in high TrkA expressing unfavourable NB.

## Introduction

The alternative TrkAIII splice variant (UniProtKB/Swiss-Prot: P04629-4) of the NGF receptor TrkA (NCBI: NM_0010122331.1; GenBank: AB019488.2; UniProtKB/Swiss-Prot: P04629) is expressed by advanced stage human neuroblastoma (NB), is associated with poor outcome in high TrkA expressing unfavourable tumours and exhibits oncogenic activity in NB models [1]–[7]. Alternative TrkAIII splicing is stress-regulated, providing a mechanism through which tumour suppressing signals from fully spliced TrkA receptors can be converted to oncogenic signals from the alternative spliced TrkAIII variant within the tumour microenvironment. We consider this to potentially represent the conservation and pathological subversion of a physiological developmental and stress-regulated, neural stem/progenitor cell stress-protection mechanism [1], [8].

Alternative TrkAIII splicing is characterised by exon 6,7 and 9 skipping and produces a TrkAIII protein that is devoid of the extracellular D4 Ig-like domain and related N-glycosylation sites required for cell surface receptor expression and prevention of ligand-independent activation [9], [10]. Unlike cell surface TrkAI (exon 9 excluded) and TrkAII (exon 9 included) splice variants [11], TrkAIII is not expressed at the cell surface but is retained within the intracellular membrane compartment, within which it exhibits spontaneous, ligand-independent activation [1]–[3]. This, results in chronic signal transduction through the IP3k/Akt/NF-κB but not Ras/MAPK pathway, which differs to activated cell surface TrkA receptors that signal also through Ras/MAPK [1], [12]–[15]. In contrast to TrkA activated at the NB cell surface, intracellular TrkAIII activity in NB cells does not inhibit proliferation nor induce neuronal differentiation but promotes an undifferentiated stem cell-like phenotype that exhibits increased tumourigenic and metastatic behaviour [1], [4]. TrkAIII exerts its “oncogenic” activity in NB cells by: protective IP3K/Akt/NF-κB signalling; induction of a pro-angiogenic pattern of gene expression; interacting with the centrosome, promoting centrosome amplification, peri-nuclear microtubule assembly and genetic instability; increasing the level of sister chromatid exchange; and modulating the unfolded protein response, pre-conditioning and adapting cells to stress [1]–[5].

Mitochondrial reactive oxygen species (ROS) also regulate stress adaptation, cellular differentiation, and chronological lifespan and play important roles in tumour pathogenesis and metastatic progression [16]–[18]. The superoxide free radical is produced during oxidative phosphorylation by single electron reduction of O_2_, leaks from respiratory chain complexes I and III and is detoxified to the non-free radical ROS H_2_O_2_ by mitochondrial superoxide dismutases (SODs), optimising physiological function [16]–[18]. Free-radical ROS do not penetrate cellular membranes but react locally and are detoxified by appropriately localised SODs. In contrast to superoxide, the non-free radical ROS H_2_O_2_ penetrates cellular membranes, acts as an extra-mitochondrial effector and is detoxified by appropriately localised catalase, glutathione peroxidase and peroxiredoxin antioxidants [17], [18]. If not tightly regulated, both free radical and non-free radical ROS cause oxidative damage to mitochondrial proteins, lipids and DNA, with fatal consequences [16], [19]–[21]. The unbridled accumulation of mitochondrial ROS represents a major mechanism of action for many chemotherapeutic agents, cytotoxic compounds and ionising radiation [20], [22], and mechanisms that attenuate the production of mitochondrial ROS promote therapeutic resistance in cancer [23]–[31].

SOD2 is the predominant mitochondrial superoxide dismutase, promotes resistance to oxygen-induced toxicity and is an absolute requirement for aerobic life [17]–[20], [24]. The *SOD2* gene, on chromosome 6, is expressed as 1.5 kb and 4.2 kb mRNAs that originate from a single promoter, differ in 3′ UTRs but encode an identical mitochondrial protein [18], [32]. SOD2 expression is regulated by CpG island methylation, histone hyper-acetylation, DNA damage and the cell cycle. SOD2 transcription is regulated by SP1, NF-κB, AP-1, AP-2, CREB, C/EBP, p53, FoxO and STAT3 transcription factors, the 4.2 kb SOD2 mRNA species predominates in undifferentiated, proliferating cells [17], [18], [32]–[34], is negatively regulated by small inhibitory RNAs of Alu/7SL origin [35] and positively regulated by SBP1 binding protein, which amplifies SOD2 transduction [36]. SOD2 enzyme activity is regulated by tyrosine nitration, serine/threonine phosphorylation and lysine acetylation [17], [18], [37]. SOD2 involvement in tumour pathogenesis and progression is controversial. Reduced SOD2 expression associates with chromosome 6 damage or deletion in some tumours, suggesting a tumour-suppressor function; defective SOD2 transcription associates with spontaneous tumourigenesis in inbred animals; tumour-associated defects in mitochondrial manganese transport reduce SOD2 activity; several tumour tissues and cell lines express low levels of SOD2; and SOD2 overexpression restores normal growth to some tumour cells [18], [32], [38], [39]. Conversely, SOD2 levels are elevated in malignant versus benign tumours; SOD2 up-regulates MMP-dependent tumour invasion and metastatic dissemination [28], [40]–[42]; SOD2 increases tumour cell resistance to ionising radiation and chemotherapy-induced toxicity [17], [18], [22], [24], [26]–[28], [31], [33], [39], [43], [44]; and higher levels of SOD2 combined with lower levels of ROS characterise normal stem and cancer stem cells and promote chemotherapeutic and radio-therapeutic resistance within the cancer stem cell niche [29], [45], [46].

SOD2 is particularly important in the nervous system. SOD2 knockout mice die within three weeks with nervous system degeneration, in association with ROS-mediated mitochondrial damage [20]. Nerve growth factor (NGF) induces SOD2 expression in PC12 cells [14], [15], [47], and promotes neuronal differentiation and survival by increasing H_2_O_2_ production, which prolongs Erk-mediated transcription, promotes Akt activation and induces formation of the cytoskeletal microtubule, mitochondria and plasma membrane complexes required for differentiation. The SOD2/ROS axis, therefore, not only modulates kinase cascades but also determines phenotype [15]. The importance of SOD2 for neuron survival is exemplified by NGF withdrawal, which reduces SOD2 expression, increases mitochondrial free-radical ROS production and induces ROS-mediated neuronal death [44], [48].

Considering the roles of SOD2 in cellular differentiation, survival, the nervous system, and in tumour pathogenesis and progression, we have assessed the influence of the TrkAIII oncogene upon SOD2 expression, activity and function in human SH-SY5Y NB cells.

## Materials and Methods

### Cell Lines and Reagents

PcDNA control, TrkAI (TrkA), TrkAIII and Y670/674/675F-mutated kinase dead (kd) TrkAIII (kd-TrkAIII) SH-SY5Y NB cell lines have been described previously [1], [2], [5]. All cell lines were grown in RPMI, supplemented with appropriate antibiotics (Zeocin for stable transfectants, penicillin and streptomycin) and 10% foetal calf serum. The TrkA inhibitors K252a [49], Gö6976 [50] and GW441756 [51], the IP3K inhibitor LY294002 [52], the MAPK inhibitor PD098059 [53], the NF-κB inhibitors PDTC [54] and Bay 11–7082 [55], the mitochondrial free radical ROS-inducing agents Rotenone [56], Paraquat [57] and LY83583 [58], Nitro blue tetrazolium and the anti-Hsp60 rabbit polyclonal antibody were purchased from Sigma-Aldrich (St. Louis Mo). The pan Trk inhibitor CEP-701 [59] was kindly supplied by Cephalon Inc. (West Chester, PA). Monoclonal anti-α-tubulin and polyclonal anti-carboxyl terminal TrkA (C14), and anti-Trx-2 (H-75) antibodies were purchased from SantaCruz (SantaCruz, Ca), the anti-phospho-Y674/675 TrkA antibody was purchased from Cell Signaling (Danvers, MA) and the mouse monoclonal anti-SOD2 antibody (anti-MnSOD) was purchased from BD Transduction Laboratories (Buccinasco, IT). Mitosox and Amplex Red were purchased from Invitrogen (Carlsbad, CA). Hybond C-extra nitrocellulose membranes and ECL solutions were purchased from Amersham International (Bedford, UK). RNA-easy RNA purification kits were purchased from Qiagen (Hilden, Ge).

### SiRNA SOD2 Knockdown

SOD2 expression was knocked down using a TriFECTa Dicer-Substrate RNAi kit employing three SOD2-specific Dicer-Substrate siRNA duplexes (duplex 1 5′-CCACUGCAAGGAACAACA-3′ and 5′-UAAGGCCUGUUGUUCCUU-3′; duplex 2∶5′-UGUAUUCAACUCGGAGAA-3′ and 5′-AGUUACAUUCUCCCAGUU-3′; duplex 3∶5′-AGUAAACCACGAUCGUUA-3′ and 5′-AUCAGCAUAACGAUCGUG-3′), as described by the manufacturer (Integrated DNA Technologies, Coralville, IA). Briefly, TrkAIII transfected SH-SY5Y cells were subjected to 48 hour transient transfection with either 25 nM negative control siRNA duplex (5′-CGUUAAUCGCGUAUAAUACGCGUAT-3′ and 5′-AUACGCGUAUUAUACGCGAUUAACGAC-3′) or 25 nM of a mix of the three SOD2 specific siRNA duplexes, using INTERFERin *in vitro* siRNA transfection reagent, as described by the manufacturer (Polyplus Transfection Inc. New York, NY). Sham transfected controls received transfection reagent alone. SiRNA knockdown of SOD2 expression was confirmed by Western blot comparison to α-tubulin in whole cell extracts (20 µg) from sham transfected, negative control siRNA-transfected and SOD2-specific siRNA transfected cells.

### Cell Extraction and Western Blotting

Cells were extracted in lysis buffer (PBS containing 0.5% sodium deoxycholate, 1% NP40, 0.1% SDS, 1 mM sodium orthovanadate, 1 mM PMSF, 1 µg/ml of pepstatin A and Aprotinin) and protein concentrations were calculated by Bradford protein concentration assay (Sigma-Aldrich). Samples were mixed with reducing SDS-PAGE sample buffer and subjected to reducing SDS-PAGE/Western blotting. Briefly, proteins separated by reducing SDS-PAGE, were trans-blotted onto Hybond C+ nitrocellulose membranes by electrophoresis (Amersham Int. UK) and the membranes subsequently air-dried. Non-specific protein binding-site on membranes were blocked by incubation for 2 hours in 5% non-fat milk in TBS-T prior to incubation with primary antibodies, at recommended dilutions, for 2–16 hours at 4°C. Membranes were then washed in TBS-T, incubated with secondary HRP-conjugated antibodies (Jackson ImmunoResearch Laboratories, West Grove, PA) diluted in blocking solution and immunoreactive species detected by chemiluminescence reaction, as directed by the manufacturer (Amersham Int).

### RNA Purification and RT-PCR

RT reactions were performed on total RNAs (1 µg), purified using RNA-easy Plus, as described by the manufacturer (Qiagen), using the Moloney Murine Leukemia virus RT kit, as detailed by the manufacturer (LifeTechnologies, Inc, Paisley, UK). RT reactions were subjected to PCR using the following primers:

SOD1∶5′-AAGGCCGTGTGCGTGCTGAA-3′ (forward primer)

5′-CAAGTCTCCAACATGCCTCT-3′ (reverse primer);

SOD2 (4.2 plus 1.5 kb mRNAs): 5′-GGAAGCCATCAAACGTGACT-3′ (forward primer)

5′-CTGATTTGGACAAGCAGCAA-3′ (reverse primer);

SOD2 (4.2 kb): 5′-AGGCAGCTGGCTCCGGTTTT-3′ (forward primer)

5′-GGCATCCCTACAAGTCCCCAAA-3′ (reverse primer);

SOD2 (1.5 kb) 5′-AGGCAGCTGGCTCCGGTTTT-3′ (forward primer)

5′-CATCAATCCCCAGCAGTGGAATAA-3′ (reverse primer);

TRX2∶5′-TTCCTGGCCCTGTCATCTC-3′ (forward primer)

5′-CTCATACTCAATGGCGAGGTC-3′ (reverse primer);

GPX: 5′-CCTCAAGTACGTCCGGCCTG-3′ (forward primer)

5′-CAACATCGTTGCGACACACC-3′ (reverse primer);

Sox2∶5′-CCAAGACGCTCATGAAGAAG-3′ (forward primer)

5′-TGGTCATGGAGTTGTACTGC-3′ (reverse primer);

CD133∶5′-GCTGATGTTGAAACTGCTTGAG-3′ (forward primer)

5′-GGTGCCGTTGCCTTGG-3′ (reverse primer);

CD117∶5′-AACGCTCGACTACCTGTGAA-3′ (forward primer)

5′-GACAGAATTGATCCGCACAG-3′ (reverse primer);

Nestin: 5′-GGATCAGATGACATTAAGACCC-3′ (forward primer)

5′-TCCAGTGGTTCTTGAATTTCC-3′ (reverse primer);

Nanog: 5′-AAGGCCGTGTGCGTGCTGAA-3′ (forward primer)

5′-CAAGTCTCCAACATGCCTCT-3′ (reverse primer);

GAPDH: 5′-AGGTCCACCACTGACACGTT-3′ (forward primer)

5′-CTGCACCACCAACTGCTTAG-3′ (reverse primer).

For each primer set, PCRs were performed on reverse transcription reactions serially diluted from 1 to 1∶1000. Reactions below saturation were compared by densitometric analysis of Jpeg images of ethidium bromide stained gels, using ImageJ64 software [60].

### Purification of Mitochondria

Mitochondria were purified using a Focus Sub cell Mitochondrial isolation kit, as described by the manufacturer (G-Biosciences, St. Louis, MO). Briefly, cells were harvested in ice cold PBS by scraping, centrifuged at 1200×g for 5 minutes, at 4°C, 500 µl of ice cold Buffer I, containing 1x protease inhibitor cocktail (Sigma), was added to the pellet and cells were disrupted by 10 passages through a 20 gauge needle. 250 µl of Buffer II was then added to the homogenate and samples were then centrifuged at 1,200×g for 5 minutes, at 4°C. Supernatants were transferred to fresh tubes, centrifuged at 15,000×g for 10 minutes, at 4°C and the resulting mitochondrial-rich pellet was washed with 500 µl of Buffer II, centrifuged at 15,000×g for 10 minutes at 4°C and re-suspended in mitochondrial storage buffer (250 mM mannitol, 5-mM HEPES (pH 7.4). Crude mitochondrial preparations in 250 mM mannitol, 5 mM HEPES (pH 7.4) and 0.5 mM EGTA were then separated by Percoll density gradient, as previously described [61]. Briefly, crude preparations were layered onto a 30% Percoll gradient, in the same buffer, and ultra centrifuged at 90,000×g for 40 minutes, at 4°C. Ultrapure mitochondria were collected from a band located 2/3rds from the top, transferred to a fresh tube, diluted 1∶10 in 250 mM mannitol, 5 mM HEPES (pH 7.4) containing 0.5 mM EGTA and re-centrifuged at 15,000×g for 10 minutes, at 4°C. Ultrapure mitochondrial pellets were re-suspended in the desired volume of cold 250 mM mannitol, 5-mM HEPES (pH 7.4) containing 0.5 mM EGTA immediately prior to experimentation.

### Mitochondrial H_2_0_2_ Assay

Mitochondrial hydrogen peroxidase (H_2_O_2_) production was measured in a fluorescence-based assay using Amplex Red (N-acetyl-3, 7-dihydroxyphenoxazine) reagent, as previously described [62]. Briefly, purified mitochondria (60 µg protein) were incubated for 10 minutes, at 37°C, in 100 µl of PBS containing 50 mU/ml of horseradish peroxidase and 40 µM Amplex Red. Fluorescence levels were monitored up to the point of stabilization (approximately 10 minutes) in a Perkin Elmer Fluoro-Count Multiwell plate reader (excitation wavelength 530 nm, emission wavelength 590 nm). Upon signal stabilization, succinate in PBS was added to a final concentration of 10 mM, reactions incubated at 37°C and fluorescence monitored at 20-minute intervals.

### Cell Death Assay

Cell death assays were performed using a modification of previously described methods [63], [64]. Briefly, cells were incubated with appropriate reagents at concentrations and for the times indicated in the results section and figure legends, washed once in Ca^2+^ free PBS, detached with ice cold PBS containing 1 mM EDTA, transferred to sterile 15 mls tubes, centrifuged for 5 minutes at 1,000×g at 4°C, washed with ice cold PBS and re-pelleted by centrifugation at 1,000×g for 5 minutes, at 4°C. Cell pellets were re-suspended in 25 µl of PBS containing 2 µl of acridine orange/ethidium bromide solution (100 µg/ml acradine orange and 100 µg/ml ethidium bromide in PBS) plated onto glass slides and examined immediately under a Zeiss “Axioplan-2” fluorescence microscope. Representative fields were digitally photographed under identical exposure conditions and the number of dead cells (orange/red nuclei) and live cells (green nuclei) counted.

### Mitosox-Red Fluorescence Detection of Free Radical ROS

Cells grown on glass chamber slide (Nunc) were treated with appropriate reagents at concentrations and for the times indicated in the results section and figure legends. Following incubation, Mitosox red solution (Invitrogen) was added to a final concentration of 2.5 µM and incubated for 10 minutes, at 37°C. Cells were then washed three times in pre-warmed PBS, mounted with a coverslip and examined immediately under a Zeiss “Axioplan-2” fluorescence microscope. Representative fields were digitally photographed under identical exposure conditions and fluorescence levels quantified from jpeg images, using ImageJ64 software [60].

### SOD Zymography

Native SOD zymography was performed as previously described [65]. Briefly, cells harvested in cold Ca^2+^ Mg^2+^-free PBS were pelleted by centrifugation at 200×g for 5 minutes, at 4°C, lysed and mitochondria ultra-purified, as described in the materials and methods section. Purified mitochondria were sonicated at 40% maximal power in a vibracell sonicator, protein concentrations determined by Bradford protein concentration assay (Sigma-Aldrich), and 150 µg of mitochondrial proteins were mixed with an equal volume of native PAGE loading buffer (1.5 M Tris Stacking gel buffer, pH 6.8, 5% bromophenol blue and 50% glycerol). Pre-prepared native 12% PAGE gels (pH 8.8), with 5% PAGE stacking gels (pH 6.8), containing sucrose and 0.004% Riboflavin-5-phopsphate to replace APS and Temed, were pre-run at 40 mA for 1 hour at 4°C to remove all traces of Temed and APS, that interfere with SOD activity, and were stored overnight at 4°C. Samples were applied to pre-prepared gels and subjected to an initial pre-electrophoresis run for three hour at 40 mA, at 8°C, to remove all remaining traces of APS and TEMED. The electrophoresis buffer was replaced and gels run for a further 2–3 hours at 40 mA at 4°C, until the bromophenol blue had reached the bottom of the gel, and then for a further 2 hours. Following electrophoresis, gels were incubated in freshly prepared SOD staining buffer (2.43 mM Nitro Blue Tetrazolium, 28 mM Temed and 0.14 mM Riboflavin in 50 mM phosphate buffer, pH 7.8) for 20 minutes in the dark, at room temperature, with shaking. Following incubation, gels were washed twice in deionized H_2_0 and exposed to UV light for 15–120 minutes, at room temperature, taking care not to let the gels dry out. Upon appearance of clear achromatic bands (SOD activity) within a dark background, gels were washed a further three times in deionized H_2_0 and left for 18–24 hours under ambient light, at room temperature, for further band development. Two achromatic bands were detected, SOD2 representing the higher and SOD1 the lower band.

### Tumour Spheroid Growth

Stable transfected SH-SY5Y cell lines were grown as tumour spheroids, as previously reported for tumour and neural stem cells spheroid growth [66]. Briefly, cells were plated in upright T25 culture flasks at a density of 1×10^5^ cells/ml in 3 ml of serum-free neuro-sphere culture medium 1∶1 DMEM/Ham’s F12 (Sigma-Aldrich), containing 0.6% Glucose, 1x N-2 and 1x B-27 nutrient supplements (Life technologies); 20 ng/ml Epidermal Growth Factor (Sigma-Aldrich), 40 ng/ml Fibroblast Growth Factor (Sigma-Aldrich), 1% glutamine and Penicillin-Streptomycin (Sigma-Aldrich). Growth factors were added every 2 days; medium was changed every 4 days and spheroid growth monitored for a period of 20 days. In SOD2 knockdown tumour spheroid assays, TrkAIII SH-SY5Y cells were transiently transfected with 25 nM control siRNA or SOD2 specific siRNA for 48 hours, as described above. At 48 hours, siRNA transfected cells were plated in neuro sphere growth medium and spheroid growth monitored for 7 and 10 days.

For the evaluation of irregular sphere volumes, digital phase contrast micrographs were made of 20 day-old tumour spheres and the three greatest spheroid dimensions (referred to as a, b and c) were used to calculate estimates of volume, using the equations for the volume of a sphere ([4 π/3]r3), where the radius r is the average of a, b and c, and the volume of an ellipsoid V = (abcπ)/6. Spherical volume calculations were based on the average radius across all three axes.

### Statistical Analysis

Data were analysed statistically by Student’s t-test and One-Way ANOVA. Statistical significance was associated with probabilities of ≤0.05.

## Results

### TrkAIII Stimulates SOD2 Expression and Mitochondrial SOD2 Activity in SH-SY5Y Cells

The effect of constitutive TrkAIII expression upon SOD2 expression and activity was compared to that of TrkA in human SH-SY5Y NB cells by Western blotting, RT-PCR and zymogram.

**Figure 1:**
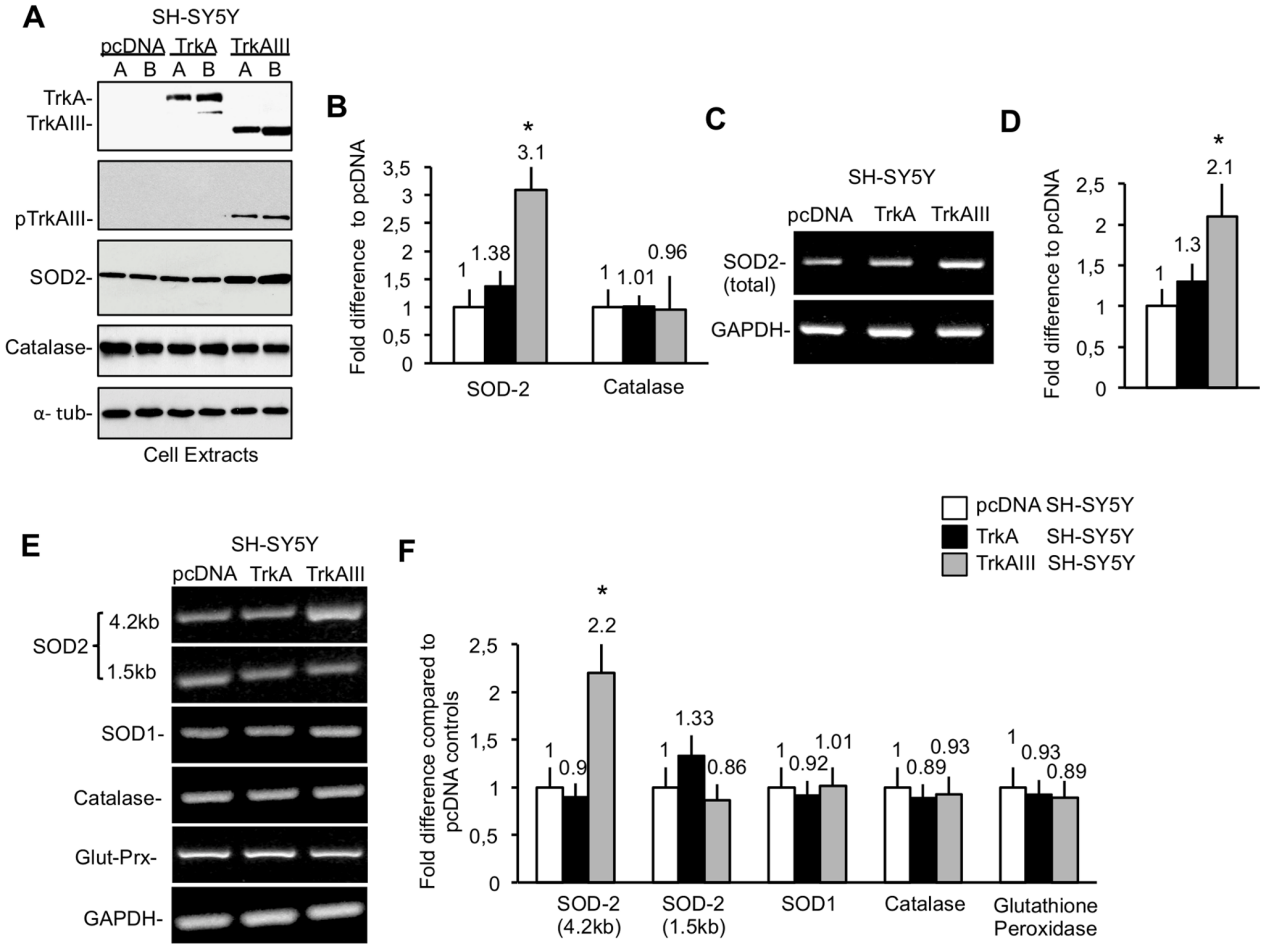
TrkAIII Augments SOD2 expression in SH-SY5Y Cells. A) Representative Western blots demonstrating the relative levels of TrkA, TrkAIII, phosphorylated TrkAIII (pTrkAIII), SOD2, catalase and α-tubulin in equal concentrations of protein extracts (20 µg) from duplicate (A, B) pcDNA, TrkA and TrkAIII SH-SY5Y cell lines. B) Histogram depicting differences in SOD2 densitometric ratio to α-tubulin between pcDNA, TrkA and TrkAIII SH-SY5Y cells. Results are displayed as the mean± s.d. fold difference in SOD2 levels, as a densitometric ratio to α-tubulin, with respect to pcDNA controls adjusted to the arbitrary value of 1±s.d., in three independent experiments. C) Representative ethidium bromide stained agarose gels demonstrating the relative levels of total (4.2 kb plus 1.5 kb) SOD2 and GAPDH RT-PCR products from selected pcDNA, TrkA and TrkAIII SH-SY5Y cell lines. D) Histogram depicting differences in SOD2 levels, as a densitometric ratio to GAPDH, in pcDNA, TrkA and TrkAIII SH-SY5Y cells. Results are displayed as the mean±s.d fold difference in SOD2 levels, as a densitometric ratio to GAPDH, with respect to pcDNA controls adjusted to the arbitrary value of 1±s.d., in 3 independent experiments performed in duplicate. E) Representative ethidium bromide stained agarose gels demonstrating relative differences in 4.2 kb SOD2, 1.5 kb SOD2, catalase, glutathione peroxidase and GAPDH RT-PCR products in selected pcDNA, TrkA and TrkAIII SH-SY5Y cell lines. F) Histogram depicting differences in 4.2 kb SOD2, 1.5 kb SOD2, SOD1, catalase and glutathione peroxidase levels, as a densitometric ratio to GAPDH, in pcDNA, TrkA and TrkAIII SH-SY5Y cell lines. Results are displayed as the mean±s.d fold difference in densitometric ratio with resect to pcDNA SH-SY5Y controls, adjusted to the arbitrary value of 1±s.d. for each ratio, in 3 independent experiments performed in duplicate. In all histograms mean values are provided above each column and asterisks denote statistical significance by t-test comparison of TrkAIII transfectants to both pcDNA and TrkA transfectants).

Western blot comparisons of total cell extract proteins (20 µg) from duplicate pcDNA control, TrkA and TrkAIII stable transfected SH-SY5Y NB cell lines revealed that TrkAIII transfectants expressed higher levels of SOD2, relative to α-tubulin, than both pcDNA control and TrkA SH-SY5Y transfectants (Fig. 1A and B). Densitometric quantification of experiments, indicated that TrkAIII transfectants exhibited a significant 3.1±0.6 fold increase (t-test, p<0.0001, degrees of freedom (df) = 10) in the SOD2 densitometric ratio to α-tubulin, when compared to pcDNA controls and a significant 2.17±0.44 fold increase (t-test, p = 0.024, df = 10) in the SOD2 densitometric ratio to α-tubulin, when compared to TrkA transfectants (data not displayed). TrkA transfectants did not exhibit a significant difference in the SOD2 densitometric ratio to α-tubulin, when compared to pcDNA controls (Fig. 1A and B). The levels of catalase in total cell extracts did not significantly differ between pcDNA, TrkA and TrkAIII SH-SY5Y transfectants (Fig. 1A and B).

In comparative semi-quantitative RT-PCR experiments, TrkAIII SH-SY5Y cells (selected clone A) exhibited a significant 2.1±0.56 fold (t-test, p = 0.02, df = 10) increase in the total (4.2 kb plus 1.5 kb) SOD2 mRNA levels, as a densitometric ratio to GAPDH, compared to pcDNA SH-SY5Y transfectants. The total SOD2 mRNA densitometric ratio to GAPDH in TrkA transfectants did not differ significantly to pcDNA controls (Fig. 1C and D).

In comparative semi-quantitative RT-PCR experiments, using primers specific for 4.2 kb and 1.5 kb SOD2 mRNAs, TrkAIII SH-SY5Y cells exhibited a significant 2.2±0.4 fold (t-test, p = 0.023, df = 10) increase in 4.2 kb SOD2 but not in 1.5 kb SOD2 levels, as a densitometric ratio to GAPDH (t-test, p = 0.12, df = 10), when compared to pcDNA SH-SY5Y controls (Fig. 1E and F). TrkA SH-SY5Y cells did not exhibit significant differences in either 4.2 kb or 1.5 kb SOD2 densitometric ratio to GAPDH, when compared to pcDNA controls (Fig. 1E and F). No significant differences were detected in SOD1, glutathione peroxidase and catalase levels, as a densitometric ratio to GAPDH, between pcDNA, TrkA and TrkAIII SH-SY5Y cells (Fig. 1E and F).

**Figure 2:**
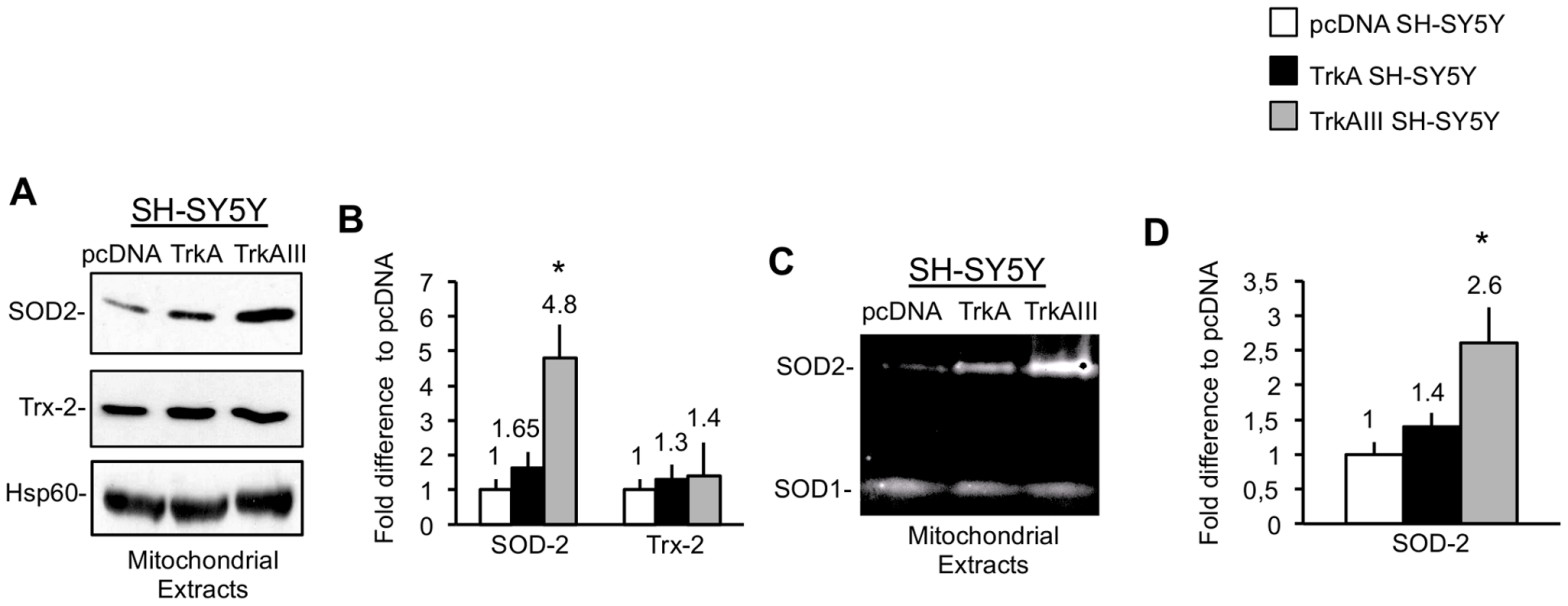
TrkAIII Augments Mitochondrial SOD-2 levels and Activity. A) Representative Western blots demonstrating relative SOD2, Trx2 and Hsp60 levels in equal protein concentrations (20 µg) of purified mitochondrial extracts from selected pcDNA, TrkA and TrkAIII SH-SY5Y cell lines. B) Histogram depicting the differences in SOD2 and Trx-2 levels, as a densitometric ratio to Hsp60, in pcDNA, TrkA and TrkAIII SH-SY5Y cell lines. Results are displayed as mean±s.d fold difference in densitometric ratio with respect to pcDNA transfectant controls adjusted to the arbitrary value of 1±s.d., in three independent experiments. C) Representative SOD activity zymogram demonstrating relative levels of SOD1 and SOD2 activity in equal protein concentrations (150 µg) of purified mitochondrial extracts from selected pcDNA, TrkA and TrkAIII SH-SY5Y cell lines. D) Histogram depicting differences in SOD2 activity levels as a densitometric ratio to SOD1 activity levels. Results are displayed as the mean±s.d fold difference in densitometric ratio with respect to pcDNA transfectant controls, adjusted to the arbitrary value of 1±s.d., in 3 independent experiments performed in duplicate. For histograms, mean values are provided above each column and asterisks denote statistical significance by t-test comparison of TrkAIII SH-SY5Y cells to either pcDNA or TrkA SH-SY5Y cells.

Densitometric analysis of Western blots, containing equal protein concentrations (20 µg) of ultra purified mitochondrial extracts from pcDNA, TrkA and TrkAIII SH-SY5Y cells, revealed a significant 4.8±0.96 fold increase (t-test, p = 0.004, df = 4) in SOD2 levels, as a densitometric ratio to Hsp60 used a mitochondrial protein loading control, in mitochondria from TrkAIII SH-SY5Y compared to pcDNA SH-SY5Y cells (Fig. 2A and B). SOD2 levels in mitochondria from TrkA SH-SY5Y cells were not significantly elevated over those detected in mitochondria from pcDNA SH-SY5Y cells (1.65±0.44 fold, t-test, p = 0.2, df = 4) (Fig. 2A and B). TrkAIII stimulation of mitochondrial SOD2 antioxidant expression was compared to that of the mitochondrial loading control Hsp60 and also the mitochondrial antioxidant thioredoxin-2 (Trx-2) [67]. Neither Hsp60 nor Trx-2 expression differed significantly in purified mitochondrial extracts from pcDNA, TrkA or TrkAIII SH-SY5Y cells (Fig. 2A and B).

Densitometric analysis of SOD activity zymograms, revealed a significant 2.6±0.52 fold increase (t-test, p = 0.018, df = 4) in SOD2 activity, measured as a densitometric ratio to SOD1 activity, in purified mitochondrial extracts (150 µg) from TrkAIII compared to pcDNA SH-SY5Y cells (Fig. 2C and D). Purified mitochondrial extracts from TrkA SH-SY5Y cells exhibited a non-significant increase in SOD2 activity, when compared to pcDNA SH-SY5Y cells (t -test, 1.4±0.2 fold, p = 0.27, df = 4) (Fig. 2C and D).

Form these data, we conclude that TrkAIII expression and spontaneous activation in SH-SY5Y cells promotes SOD2 mRNA and protein expression, augments mitochondrial SOD2 but not Trx2 levels and increases mitochondrial SOD2 activity.

### TrkA, IP3K and NF-κB Inhibitors Reduce SOD2 Expression in TrkAIII Transfectants

In order to further characterise signalling pathways involved in TrkAIII promotion of SOD2 expression, experiments were performed under TrkA, IP3K, MAPK and NF-κB inhibitory conditions. Wild type (wt) TrkAIII was also compared to tyrosine kinase dead (kd) Y670/674/675F-mutated TrkAIII.

**Figure 3:**
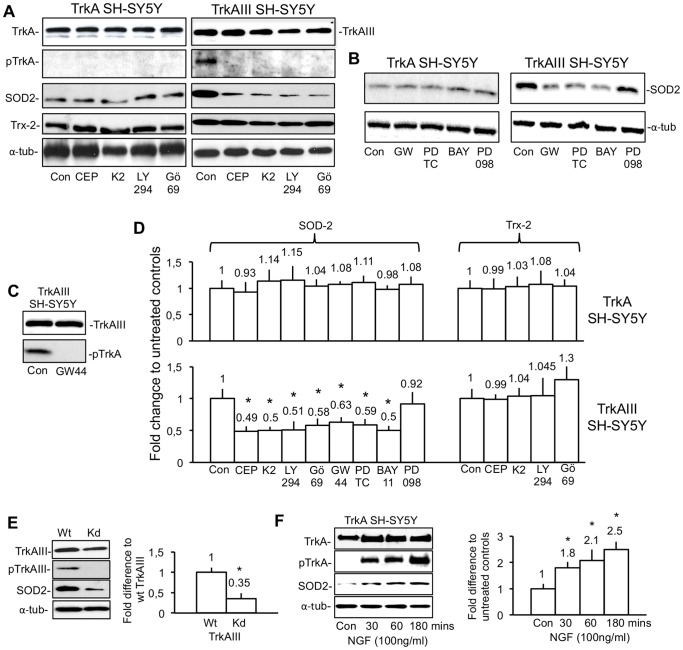
SOD2 expression in TrkAIII SH-SY5Y cells is reduced by TrkA, IP3K and NF-κB inhibitors. A) Western blots demonstrating relative differences in TrkA, TrkAIII, phosphorylated TrkA (pY674/675-TrkA), SOD2, Trx-2 and α-tubulin levels in equal protein concentrations (20 µg) of total extracts from TrkA and TrkAIII SH-SY5Y cell lines, treated without (Con) or with 1 µM CEP-701 (CEP), 1 µM K252a (K2); 25 µM LY294002 (LY294) or 1 µM Gö6976 (Gö69) for 24 hours, at 37°C. B) Representative Western blots demonstrating relative differences in SOD2 and α-tubulin levels in equal protein concentrations (20 µg) of total extracts from TrkA and TrkAIII SH-SY5Y cell lines treated without (Con) or with: 100 nM GW441756 (GW44), 500 µM PDTC (PDTC), 100 µM Bay 11–7082 (BAY11) or 30 µM PD098059 (PD098). C) Representative Western blot of total cell extracts (20 µg), demonstrating inhibition of constitutive TrkAIII Y674/675 phosphorylation (Con) following 3 hours treatment of TrkAIII transfectants with 100 nM GW441756 (GW44). D) Histograms showing the densitometric differences in the SOD2 and Trx-2 levels, as a ratio to α-tubulin, in equal concentrations (20 µg) of total cell extracts from TrkA and TrkAIII SH-SY5Y cells treated without (Con) or with: 1 µM CEP-701 (CEP); 1 µM K252a (K2); 25 µM LY294002 (LY294); 1 µM Gö6976 (Gö69); 100 nM GW441756 (GW44); 500 µM PDTC (PDTC); 100 µM Bay 11–7082 (BAY11) or 30 µM PD098059 (PD098), for 24 hours at 37°C. Results are displayed as mean±s.d fold change compared to untreated controls, adjusted to the arbitrary value of 1±s.d., in 3 independent experiments. Mean values are presented above each column and asterisks denote statistical significance by t-test comparison of treated and untreated TrkAIII SH-SY5Y cells. E) Representative Western blots demonstrating differences in TrkAIII, phosphorylated TrkAIII (pTrkAIII), SOD2 and α-tubulin levels in equal concentrations (20 µg) of total cell extracts from wt-TrkAIII and kd-TrkAIII SH-SY5Y cell lines plus a histogram depicting the densitometric difference in the SOD2 levels as a ratio to α-tubulin, in total cell extracts from wt-TrkAIII and kd-TrkAIII SH-SY5Y cells, displayed as mean±s.d fold difference in densitometric ratio with respect to wt-TrkAIII transfectants adjusted to the arbitrary value of 1±s.d., in three independent experiments. Mean values are presented above each column and asterisks denote statistical significance by t-test comparison of wt and kd TrkAIII transfectants. F) Representative Western blots demonstrating levels of TrkA, tyrosine phosphorylated TrkA (pTrkA), SOD2 and α-tubulin in equal concentrations (20 µg) of total cell extracts from untreated TrkA SH-SY5Y cells (Con) and TrkA SH-SY5Y cells treated for 30, 60 and 180 minutes with 100 ng/ml NGF, plus a histogram showing densitometric differences in the SOD2: α-tubulin ratio in total cell extracts from untreated (Con) and NGF-treated TrkA SH-SY5Y cells (100 ng/ml for 30, 60 and 180 minutes), displayed as mean±s.d. fold difference in SOD2 levels with respect to untreated controls adjusted to the arbitrary value of 1±s.d., in 3 independent experiments. Mean values are provided above each column and asterisks denote statistical significance by t-test comparison between NGF-treated and untreated TrkA SH-SY5Y controls.

Densitometric analysis of Western blots, containing equal protein concentrations (20 µg) of total cell extracts from TrkA SH-SY5Y cells, treated for 24 hours with or without (Con) 1 µM CEP-701, 1 µM K252a, 1 µM Gö6976, 100 nM GW441769, 25 µM LY294002, 30 µM PD098059, 500 µM PDTC and 100 µM Bay 11–7082, revealed no significant alteration in SOD2 or Trx2 levels, as a densitometric ratio to α-tubulin (Fig. 3A and B). In TrkAIII SH-SY5Y cells, SOD-2 levels were significantly reduced, as a densitometric ratio to α-tubulin, by 51.1±7.6% (t-test, p = 0.01, df = 4) following treatment with 1 µM CEP-701; 50.3±12% (t-test, p = 0.01, df = 4) following treatment with 1 µM K252a; 49.2±13.3% (t-test, p = 0.01, df = 4) following treatment with 25 µM LY294002; 42.4±18.3% (t-test, p≤0.01, df = 4) following treatment with 1 µM Gö6976; 37±12.5% (t-test, p≤0.01, df = 4) following treatment with 100 nM GW441769; 41+15.4% (t-test, p = 0.04, df = 4) following treatment with 500 µM PDTC; and by 50.2±14.4% following treatment with 100 µM Bay 11–7082 (t-test, p = 0.01, df = 4), but were not significantly reduced following treatment with 30 µM PD098059 (Fig. 3B and D). Constitutive Y674/675 (pY674/675) TrkAIII tyrosine kinase loop phosphorylation was abrogated following treatment with CEP-701, K252a, Gö6976 and LY294002, under these conditions (Fig. 3A). GW441769 (100 nM) abrogated TrkAIII pY674/675 tyrosine phosphorylation, following 3 hours incubation with TrkAIII SH-SY5Y cells (Fig. 3C).

Densitometric comparison of Western blots, containing equal protein concentrations (20 µg) of total cell extracts from wt-TrkAIII and kd-TrkAIII stable SH-SY5Y transfectants, revealed significant 2.86±0.34 fold (t-test, p = 0.001, df = 4) higher levels of SOD2 protein, as a densitometric ratio to α-tubulin, in wt-TrkAIII compared to kd-TrkAIII SH-SY5Y cells (65% reduced levels of SOD2) (Fig. 3E).

Densitometric comparison of Western blots, containing equal protein concentrations (20 µg) of total cell extracts from untreated (Con) and NGF (100 ng/ml)-treated TrkA SH-SY5Y cells, revealed a significant increase in SOD2 levels, as a densitometric ratio to α-tubulin, at 30 minutes (1.8±0.23 fold increase, t-test, p = 0.007, df = 4), 60 minutes (2.1±0.42 fold increase, t-test, p = 0.013, df = 4) and 180 minutes (2.5±0.29 fold increase, t-test, p = 0.001, df = 4) following NGF addition (Fig. 3F).

**Figure 4:**
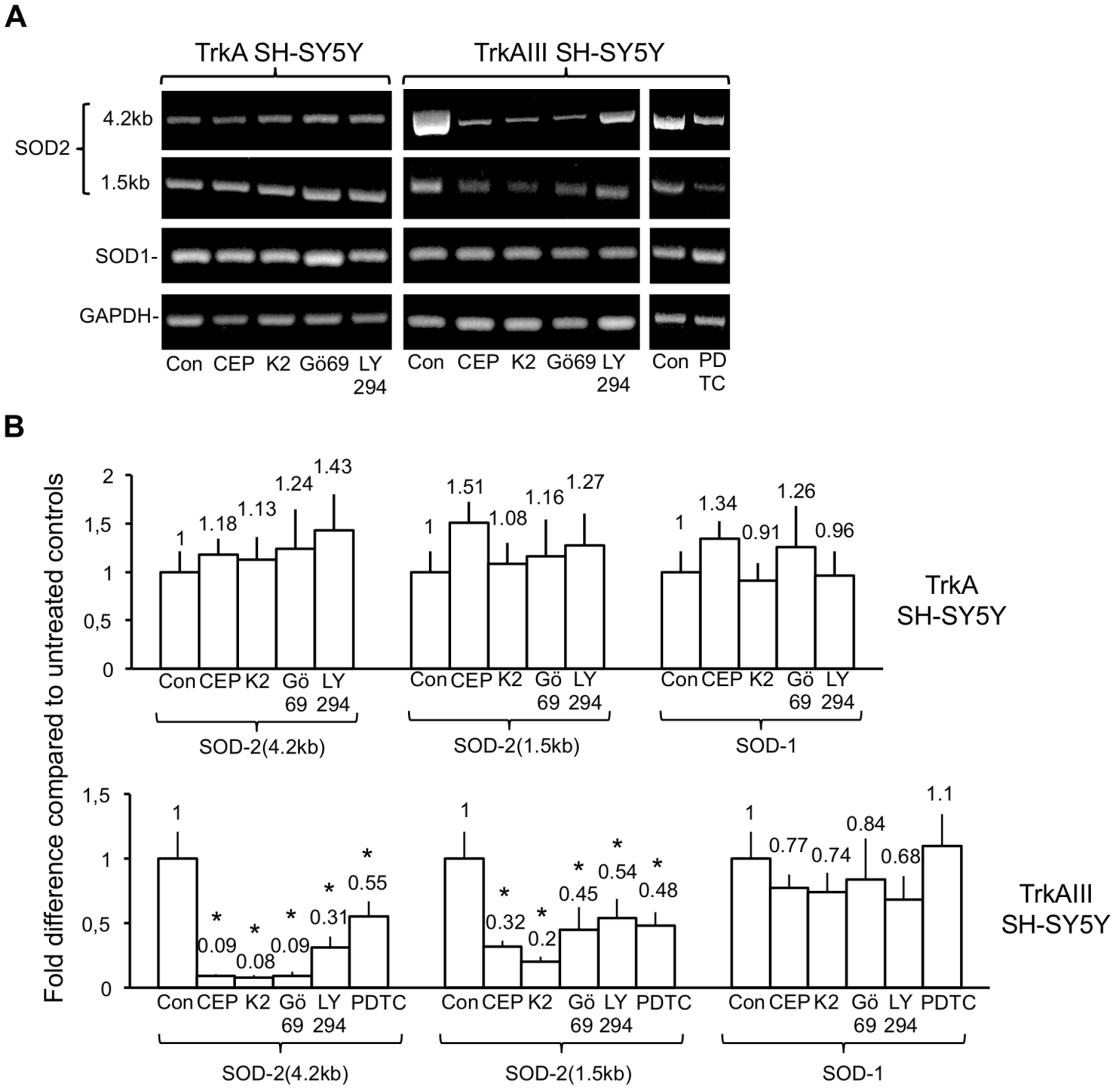
SOD2 mRNA expression in TrkAIII SH-SY5Y cells is reduced by TrkA, IP3K and NF-κB inhibitors. A) Representative ethidium bromide stained agarose gels demonstrating differences in 4.2 kb SOD2, 1.5 kb SOD2, SOD1 and GAPDH RT-PCR levels in TrkA SH-SY5Y and TrkAIII SH-SY5Y cells, treated without (Con) or with: 1 µM CEP-701 (CEP); 1 µM K252a (K2); 1 µM Gö6976 (Gö69) or 25 µM LY294002 (LY294) or 10 µM PDTC (PDTC) for 12 hours at 37°C. B) Histograms showing fold difference to untreated controls of 4.2 kb SOD2, 1.5 kb SOD2 and SOD1 RT-PCR products, as ratios to GAPDH, in TrkA SH-SY5Y and TrkAIII SH-SY5Y cells treated without (Con) or for 12 hours, at 37°C, with: 1 µM CEP-701 (CEP); 1 µM K252a (K2); 1 µM Gö6976 (Gö69); 25 µM LY294002 (LY294); or 10 µM PDTC (PDTC). Results are displayed as mean±s.d fold difference compared to untreated controls (Con), each adjusted to the arbitrary value of 1±s.d., in 3 independent experiments performed in duplicate. Mean values are provided above each column and asterisks denote statistical significance by t-test comparison of treated and untreated TrkA and TrkAIII SH-SY5Y cells, respectively.

Semi-quantitative RT-PCR experiments revealed that TrkA SH-SY5Y cells treated for 12 hours with 1 µM CEP-701, 1 µM K252a, 1 µM Gö6976 or 25 µM LY294002 did not exhibit significant differences in either 4.2 kb SOD2, 1.5 kb SOD2 or SOD1 mRNA levels, as a densitometric ratio to GAPDH RT-PCR levels, when compared to untreated TrkA SH-SY5Y controls (Fig. 4A and B). In TrkAIII SH-SY5Y cells, 4.2 kb SOD2 mRNA levels as a densitometric ratio to GAPDH, exhibited a significant 75±10.5% (t-test, p = 0.004, df = 4) reduction following overnight (16 hr) treatment with 1 µM CEP-701; an 82±16.4% reduction (t-test, p = 0.003, df = 4) following overnight (16 hr) treatment with 1 µM K252a; an 85±32.3% reduction (t-test, p = 0.002, df = 4) following overnight (16 hr) treatment with 1 µM Gö6976; a 60±16.2% reduction (t-test, p = 0.01, df = 4) following overnight (16 hr) treatment with 1 µM LY294002 and a 45.2+9.9% reduction (t-test, p = 0.015, df = 4) following overnight (16 hr) treatment with 10 µM PDTC, when compared to untreated TrkAIII SH-SY5Y controls. Furthermore, 1.5 kb SOD2 mRNA levels in TrkAIII transfectants were also significantly reduced as a densitometric ratio to GAPDH by 55.2±7.7% (t-test, p = 0.01, df = 4) following CEP-701 treatment; 70±14% (t-test, p = 0.005, df = 4) following K252a treatment; 52.2±19.8% (t-test, p≤0.03, df = 4) following Gö6976 treatment; 42.1±9.8% (t-test, p = 0.04, df = 4) following LY294002 treatment and 52.3±13% (t-test, p = 0.03, df = 4) following PDTC treatment, when compared to untreated TrkAIII SH-SY5Y controls. Overnight treatment with CEP-701, K252a, Gö6976, LY294002 and PDTC did not reduce SOD1 mRNA levels in TrkAIII SH-SY5Y cells (Fig. 4A and B).

From these data, we conclude that TrkAIII tyrosine kinase activity is required to stimulate SOD2 expression in SH-SY5Y cells, that this involves IP3K and NF-κB but not MAPK.

### TrkAIII Attenuates Mitosox-reactive Mitochondrial Free-radical ROS Production

Since mitochondrial SOD2 activity regulates free radical ROS accumulation, mitochondrial free radical ROS production in response to the free radical ROS inducers Rotenone [56], Paraquat [57] and LY83583 [58] was compared in pcDNA control, TrkA and TrkAIII SH-SY5Y cells, in a Mitosox-based assay.

**Figure 5:**
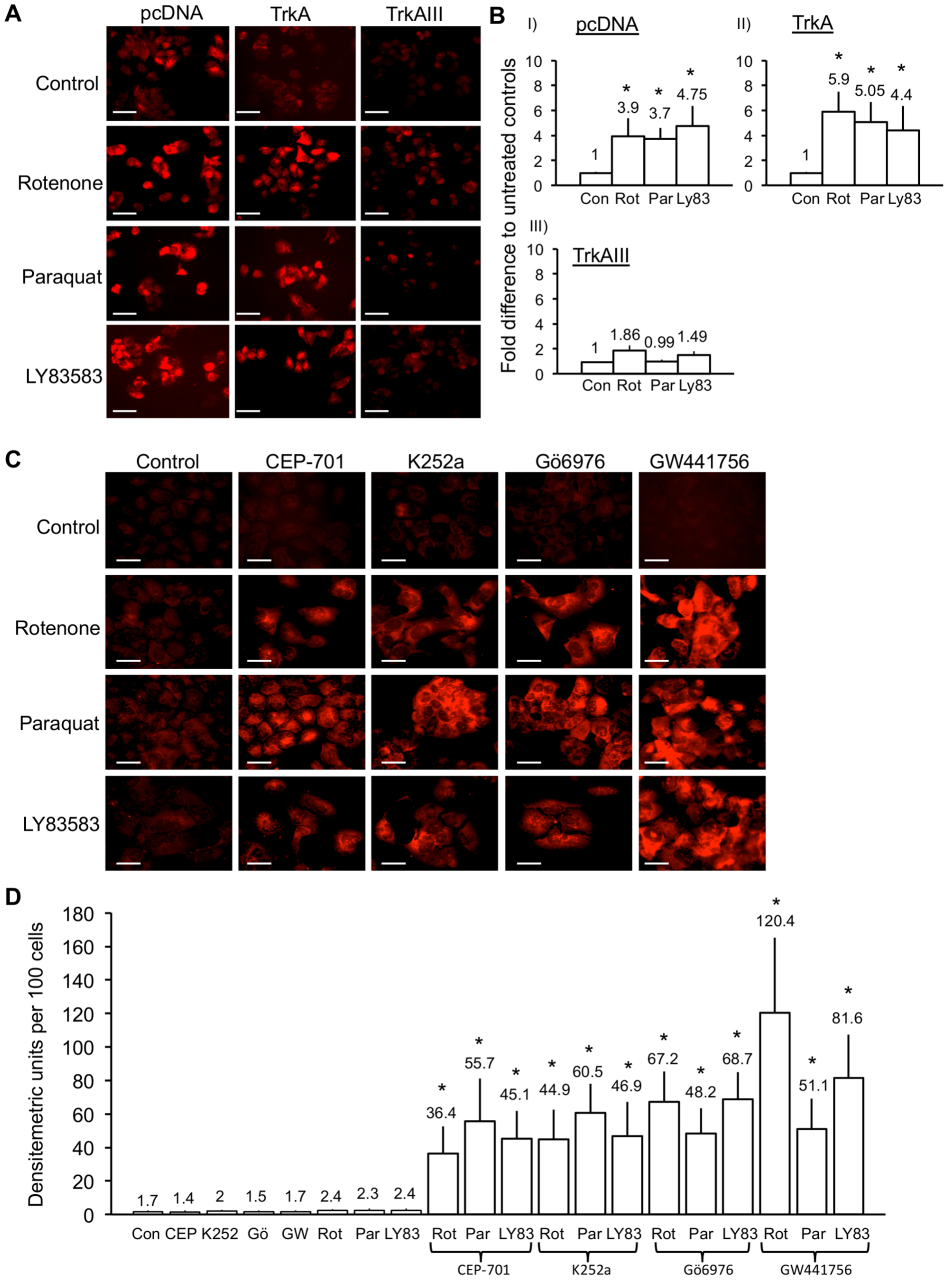
TrkAIII attenuation of mitochondrial ROS production is reversed by TrkA inhibitors. A) Digital fluorescence micrographs demonstrating Mitosox-reactive ROS (red) in untreated pcDNA, TrkA and TrkAIII SH-SY5Y cell lines (Control) or following treatment for 12 hours, at 37°C with: 1 µM Rotenone; 250 µM Paraquat; or for 3 hours at 37°C with 1 µM LY83583 (bar = 100 µm). B) Histograms showing densitometric fold difference in Mitosox reactive ROS levels in pcDNA, TrkA and TrkAIII SH-SY5Y cells following 12 hours treatment with 1 µM Rotenone (Rot); for 12 hours with 250 µM Paraquat (Par); or for 3 hours with 1 µM LY83583, compared to untreated controls (Con), given the arbitrary value of 1±s.d. Results are displayed as mean±s.d fold difference compared to untreated controls in three independent experiments. Mean values are provided above each column and asterisks denote statistical significance by t-test comparison of treated cell lines to untreated controls. C) Digital fluorescence micrographs demonstrating Mitosox-reactive ROS (red) in untreated TrkAIII SH-SY5Y cells (Control); TrkAIII SH-SY5Y cells treated for 36 hours with 1 µM CEP-701, 1 µM K252a, 1 µM Gö6976 or 100 nM GW441756, alone; TrkAIII SH-SY5Y cells treated for 12 hours with either 1 µM Rotenone, 250 µM Paraquat or for 3 hours with 1 µM LY83583, alone; and TrkAIII SH-SY5Y cells pre-incubated for 24 hours with either 1 µM Cep-701, 1 µM K262a, 1 µM Gö6976 or 100 nM GW441756, then treated for 12 hours with either 1 µM Rotenone, 250 µM Paraquat or for 3 hours with 1 µM LY83583, in the continuous presence of each respective inhibitor (bar = 100 µm). D) Histogram demonstrating the differences in ROS levels detected in untreated TrkAIII SH-SY5Y cells (Con); TrkAIII SH-SY5Y cells treated with 1 µM CEP-701 (CEP), 1 µM K252a (K252), 1 µM Gö6976 (Gö69), 100 nM GW441756 (GW4), 1 µM Rotenone (Rot), 250 µM Paraquat (Par) or 1 µM LY83583 (LY83), alone; or with 1 µM Rotenone (Rot), 250 µM Paraquat (Par) or 1 µM LY83583 (LY83), following pre-incubation with, and in the continuous presence of, 1 µM CEP-701, 1 µM K252a, 1 µM Gö6976 or 100 nM GW441756, as described for 5C. Results are expressed as mean±s.d densitometric units per 100 cells, in three independent experiments performed in duplicate. Mean values are provided above each column and asterisks denote statistical significance by Student’s t-test (p<0.0004, df = 10) and One-Way ANOVA ((2, 15) P<0.0001), comparing inhibitor alone, ROS-inducer alone and inhibitor plus ROS-inducer groups.

Densitometric comparisons of fluorescent micrographs adjusted for cell number, revealed that Mitosox-reactive mitochondrial ROS levels in pcDNA SH-SY5Y cells were significantly increased by 3.9±1.5 fold (t-test, p = 0.0008, df = 10) following 6 hour treatment with 1 µM Rotenone; 3.7±0.9 fold following 6 hour treatment with 250 µM Paraquat (t-test, p<0.0001, df = 10) and 4.75±1.6 fold (t-test, p = 0.0002, df = 10) following 3 hour treatment with 1 µM LY83583. In TrkA SH-SY5Y cells, free radical ROS levels were significantly increased by: 5.9±1.6 fold (t-test, p<0.0001, df = 10) following 6 hour treatment with 1 µM Rotenone; 5.05±1.6 fold (t-test, p = 0.0001, df = 10) following 6 hour treatment with 250 µM Paraquat; and 4.4±1.95 fold (t-test, p<0.0016, df = 10) following 3 hour treatment with 1 µM LY83583. In contrast, mitochondrial free radical ROS levels in TrkAIII SH-SY5Y cells were not significantly increased by treatment with Rotenone, Paraquat or LY83583 (Fig. 5A and B).

In inhibitor studies, densitometric analysis of free radical ROS levels adjusted to densitometric units per 100 cells, revealed no significant increase in TrkAIII SH-SY5Y cells following 27 or 48 hours treatment with either 1 µM CEP-701, 1 µM K252a, 1 µM Gö6976 or 100 nM GW441756, alone (Fig. 5B and C, results displayed for 48 hours treatment only).

As described above, treatment of TrkAIII SH-SY5Y cells with the free radical ROS inducers Rotenone, Paraquat and LY83583 did not significantly increase the ROS levels compared to mock-treated controls. However, TrkAIII SH-SY5Y cells produced significant levels of ROS in response to these ROS inducers when cells were pre-treated with the TrkA tyrosine kinase inhibitors CEP-701, K252a, Gö6976 or GW441756. These tyrosine kinase inhibitors by themselves did not affect ROS levels in TrkAIII SH-SY5Y cells (Fig. 5C and 5D). From these data, we conclude that TrkAIII tyrosine kinase activity is necessary to inhibit mitochondrial ROS production induced by Rotenone, Paraquat or LY83583.

### SOD2 siRNA Restores the Sensitivity of TrkAIII SH-SY5Y Cells to Rotenone, Paraquat and LY83583-induced Mitosox-reactive Mitochondrial Free-radical ROS Production

In order to confirm SOD2 involvement in the reduced sensitivity of TrkAIII SH-SY5Y cells to agents that induce mitochondrial free radical ROS, the induction of mitochondrial ROS was assessed in TrkAIII SH-SY5Y cells exhibiting siRNA knockdown of SOD2 expression.

**Figure 6:**
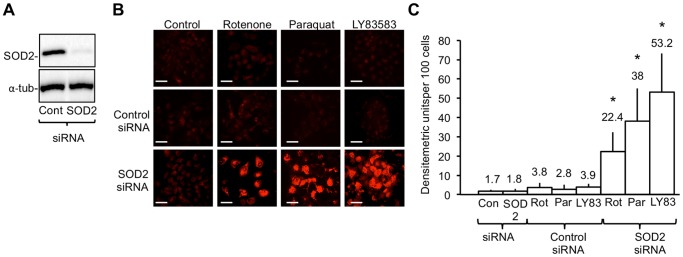
SiRNA SOD2 knockdown restores the sensitivity TrkAIII SH-SY5Y to agents that induce mitochondrial ROS production. A) Western blot demonstrating knockdown of SOD2 but not α-tubulin protein expression in TrkAIII SH-SY5Y cells transiently transfected for 48 hours with SOD2-specific but not with control siRNA, prior to assay. B) Digital fluorescence micrographs demonstrating Mitosox-reactive ROS (red) in TrkAIII SH-SY5Y cells cultured in the absence of siRNAs or ROS-inducing reagents (Control); treated with 25 nM control SiRNA or 25 nM SOD2 siRNA alone; or treated with either 25 nM control SiRNA or 25 nM SOD2 siRNA followed by 12 hours with 1 µM Rotenone or 250 µM Paraquat, or 3 hours with 1 µM LY83583 (bar = 100 µm). C) Histogram demonstrating differences in ROS levels in TrkAIII SH-SY5Y cells treated with control 25 nM siRNA-treated (Con SiRNA) or 25 nM SOD2 siRNA, alone; or for 12 hours with 1 µM Rotenone (Rot), 250 µM Paraquat or for 3 hours with 1 µM LY83583 (LY835), following 48 hours treatment with either control siRNA or SOD2 siRNA. Results are expressed as mean±s.d densitometric units per 100 cells, in three independent experiments performed in duplicate. Mean values are provided above each column and asterisks denote statistical significance by Student’s t-test (p<0.0005, df = 10) and One-Way ANOVA ((2, 15) p<0.0001), comparing inhibitor alone, ROS-inducer alone and inhibitor plus ROS-inducer groups.

In Western blots, whole cell extracts from TrkAIII SH-SY5Y cells treated for 48 hours SOD2-specific but not negative control siRNA duplexes exhibited a significant reduction in SOD2 compared to α-tubulin protein levels, used as a loading control (Fig. 6A).

SiRNA knockdown of SOD2 expression in TrkAIII SH-SY5Y cells, but not transfection with control siRNA, resulted in the production of significant levels of ROS in response to Rotenone, Paraquat and LY83538 (Figs. 6B and 6C). From these results we conclude that elevated SOD2 expression in TrkAIII SH-SY5Y cells is largely responsible for attenuating Rotenone, Paraquat, and LY83583-induced mitochondrial ROS production.

### TrkAIII Promotes H_2_0_2_ Production by Purified Mitochondria

Since, mitochondrial SOD2 regulates H_2_0_2_ production, H_2_0_2_ production was compared in highly purified mitochondria from pcDNA control, TrkA and TrkAIII transfected SH-SY5Y cells.

**Figure 7:**
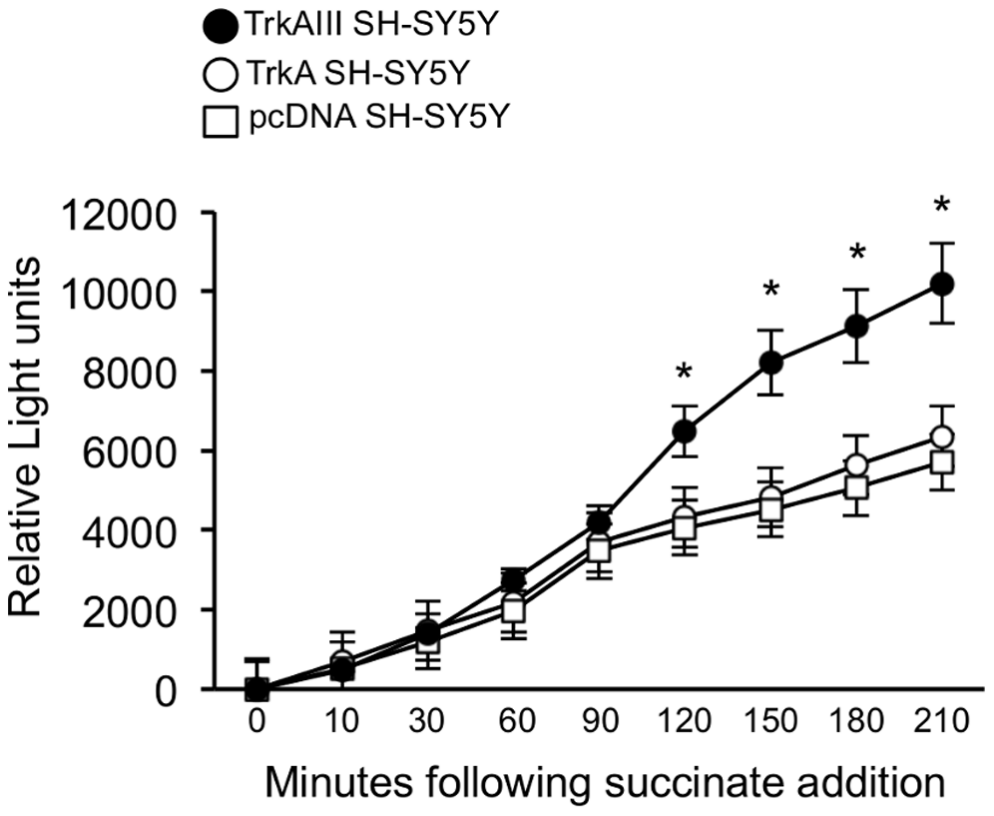
TrkAIII promotes mitochondrial hydrogen peroxide production. Line graph displaying comparative levels of H_2_0_2_ produced over a 210-minute time course by equivalent protein concentrations (60 µg) of intact purified mitochondria from pcDNA, TrkA and TrkAIII SH-SY5Y cells, in an Amplex Red-based assay, following reaction initiation with 10 mM succinate. Results are displayed as the mean±s.d. relative light units produced in minutes following 10 mM succinate addition, in three independent experiments performed in duplicate (asterisks denote statistical significance by t-test comparison of TrkAIII SH-SY5Y cells to either pcDNA or TrkA SH-SY5Y cells).

In mitochondrial H_2_0_2_ production assays, purified mitochondria (60 µg) from TrkAIII SH-SY5Y cells produced significantly more H_2_0_2_ at 120 to 210 minutes following reaction initiation by succinate addition, when compared to pcDNA SH-SY5Y and TrkA SH-SY5Y cells (Fig. 7). In comparison to H_2_0_2_ standards, mitochondria from pcDNA SH-SY5Y cells produced a mean of 4.92±2.96 pMoles H_2_0_2_/µg protein/minute, mitochondria from TrkA SH-SY5Y cells produced a mean of 5.8±3.88 pMoles H_2_0_2_/µg protein/minute and mitochondria from TrkAIII SH-SY5Y cells exhibited a significantly higher rate of H_2_0_2_ production of 14.07±3.88 pMoles H_2_0_2_/µg protein/minute (t-test, p = 0.001, df = 10, for TrkAIII versus pcDNA and p = 0.004, df = 10 for TrkAIII versus TrkAI SH-SY5Y cells).

From these data, we conclude that TrkAIII SH-SY5Y cells produce higher levels of H_2_0_2_ compared to controls in response to succinate, presumably due to elevated SOD2 activity.

### TrkAIII Promotes Growth of Tumour Spheroids that Express Stem Cell Markers

Since, spheroidal growth under defined conditions is a characteristic of neural cancer stem cells [66]; cancer stem cells express higher levels of SOD2 than non-stem cell counterparts [45], [46]; TrkAIII promotes a more stem cell-like phenotype [1], [4]; and NB staminality associates with increased CD133, CD117, SOX2, Nestin and Nanog expression [66], [68], [69], spheroid growth capacity and the expression of SOD2, CD133, CD117, SOX2, Nestin and Nanog were compared in pcDNA control, TrkA and TrkAIII SH-SY5Y cells.

PcDNA control, TrkA and TrkAIII SH-SY5Y cells all grew as tumour spheroids under defined spheroid growth conditions [66].

**Figure 8:**
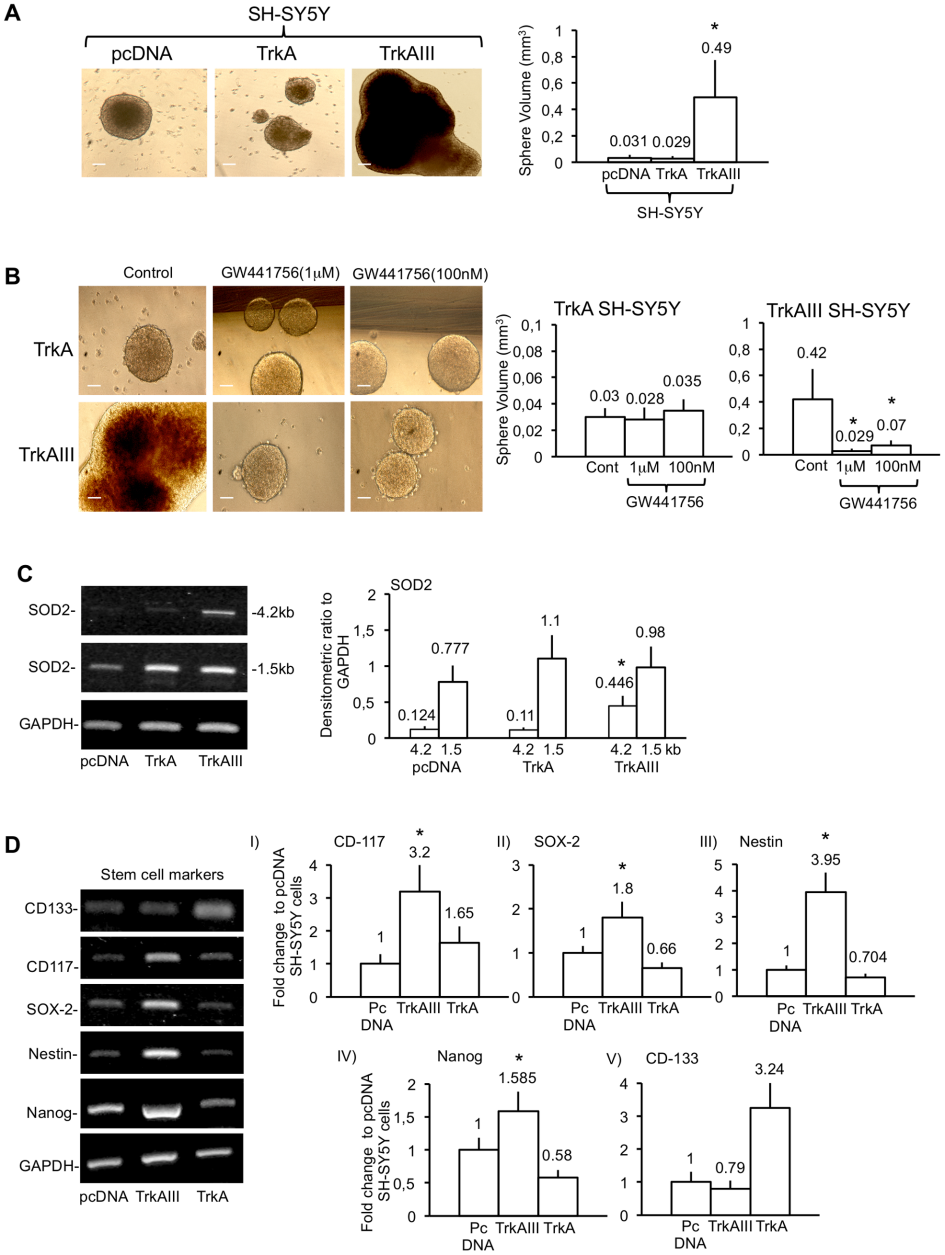
TrkAIII promotion of SH-SY5Y spheroid growth is inhibited by GW441756 and associates with stem cell marker expression. A) Representative digital phase-contrast micrographs, demonstrating differences in the relative size of 20-day pcDNA, TrkA and TrkAIII SH-SY5Y tumour spheroids (bar = 100 µm), plus a histogram showing the difference in 20-day pcDNA, TrkA and TrkAIII SH-SY5Y tumour spheroid volumes displayed as the mean+s.d. volume (mm^3^) of 50 individual tumour spheres per cell line, in three independent experiments. Mean values are provided above each histogram column and asterisks denote statistical significance by t-test comparison of TrkAIII SH-SY5Y cells to either pcDNA or TrkA SH-SY5Y cells. B) Digital phase-contrast micrographs demonstrating differences in the relative tumour spheroid sizes in TrkA and TrkAIII SH-SY5Y cells cultured for 20 days in the absence (Control) or presence of 100 nM or 1 µM GW441756 (bar = 100 µm), plus a histogram showing the difference in TrkA and TrkAIII SH-SY5Y tumour spheroid volumes, cultured as described above, displayed as the mean+s.d. volume (mm^3^) of 50 individual tumour spheres per cell line, in three independent experiments. Mean values are provided above each histogram column and asterisks denote statistical significance by t-test comparison of GW441756-treated and untreated TrkAIII SH-SY5Y cells. C) Ethidium bromide stained agarose gels demonstrating the relative levels of 4.2 kb SOD2, 1.5 kb SOD2 and GAPDH RT-PCR products in 20-day pcDNA, TrkA and TrkAIII SH-SY5Y spheroids, plus a histogram depicting the relative densitometric differences in 4.2 kb SOD2 and 1.5 kb SOD2 mRNA expression. Results are displayed as mean+s.d. densitometric ratio to GAPDH, in three independent experiments. Mean values are provided above each histogram column and asterisks denote statistical significance by t-test comparison of TrkAIII to both pcDNA and TrkA SH-SY5Y cells. D) Ethidium bromide stained agarose gels demonstrating differences in CD133, CD117, SOX-2, Nestin, Nanog and GAPDH RT-PCR products in 20-day pcDNA, TrkA and TrkAIII SH-SY5Y spheroids, plus histograms depicting fold change in (I), CD117, (II) SOX-2, (III) Nestin, (IV) Nanog and (V) CD133 expression in 20-day tumour spheroids. Results are displayed as mean±s.d. fold change compared to pcDNA SH-SY5Y cells, given the arbitrary value of 1 as a densitometric ratio to GAPDH expression, in three independent experiments. Mean values are provided above each histogram column and asterisks denote statistical significance by t-test comparison of TrkAIII to pcDNA SH-SY5Y cells.

PcDNA, TrkA and TrkAIII SH-SY5Y cells plated at a density of 1×10^5^ cells/ml, formed tumour spheroids within 7 days. At 20 days, tumour spheroids from TrkAIII transfectants exhibited a significant 15.8±9.9 fold (t-test, p = 0.001, df = 4) larger mean volume than pcDNA transfectants and a significant 16.9±9.8 fold (t-test, p = 0.001, df = 4) larger mean volume than TrkA transfectants. At 20 days, TrkA and pcDNA tumour spheroids did not differ significantly in mean volume (Fig. 8A).

In inhibitor studies, K252a (1 µM) and CEP-701 (1 µM) completely abrogated spheroid growth capacity of pcDNA, TrkA and TrkAIII SH-SY5Y cells (data not displayed). In contrast, the TrkA specific inhibitor GW441756, at concentrations of 1 µM and 100 nM, did not significantly inhibit spheroid growth of TrkA SH-SY5Y cells but significantly reduced tumour spheroid growth of TrkAIII SH-SY5Y cells, reducing mean spheroid volume from 0.42±0.23 mm^3^ (control) to 0.029±0.014 mm^3^ (t-test, p<0.05, df = 4) with 1 µM GW441756 and 0.05±0.015 mm^3^ with 100 nM GW441756 (t-test, p<0.05, df = 4; One way ANOVA comparison: F (2, 6) = 8.16; p<0.019) (Fig. 8B). Mean spheroid volumes of GW441756-treated TrkAIII SH-SY5Y cells did not differ significantly to those of GW441756-treated TrkA SH-SY5Y cells (Fig. 8B).

In semi quantitative RT-PCR assays, densitometric comparison of mRNAs purified from 20-day spheroids revealed a significant 3.6±1.6 fold (t-test, p = 0.0002, df = 10) increase in 4.2 kb but not 1.5 kb SOD2 mRNA levels in TrkAIII SH-SY5Y spheroids compared pcDNA SH-SY5Y spheroids, as a densitometric ratio to GADPH mRNA levels (Fig. 8C). TrkAIII SH-SY5Y spheroids also exhibited a significant 3.2±0.8 (t-test, p = 0.0003, df = 10) fold higher level of CD117 (Fig. 8D(I)); 1.8±0.37 (t-test, p<0.0001, df = 10) higher level of SOX2 (Fig. 8D(II)); 3.95±0.73 (t-test, p<0.0001, df = 10) fold higher level of Nestin (Fig. 8D(III)); and 1.56±0.3 fold (t-test, p<0.002, df = 10) higher level of Nanog (Fig. 8D(IV)) than pcDNA SH-SY5Y spheroids (Fig. 8D); and a 2.75±0.48 (t-test, p<0.0001, df = 10) fold higher level of SOX2 (Fig. 8D(II)); 5.5±0.86 (t-test, p = 0.0001, df = 10) fold higher level of Nestin (Fig. 8D(III)), 2.74±0.58 fold (t-test, p<0.0001, df = 10) higher level of Nanog (Fig. 8D(IV)); a 78±25.3% reduction in the level of CD133 (Fig. 8D(V)) and a non-significant 1.8 fold increase in the level of CD117, when compared to TrkA SH-SY5Y spheroids, as a densitometric ratio to GAPDH (Fig. 8D(I)). CD133 levels were low in all tumour spheroids and detected only in undiluted RT reactions, following 40 cycles of RT-PCR. Despite this, CD133 levels were higher in TrkA compared to both pcDNA and TrkAIII SH-SY5Y spheroids (Fig. 8D(V)).

**Figure 9:**
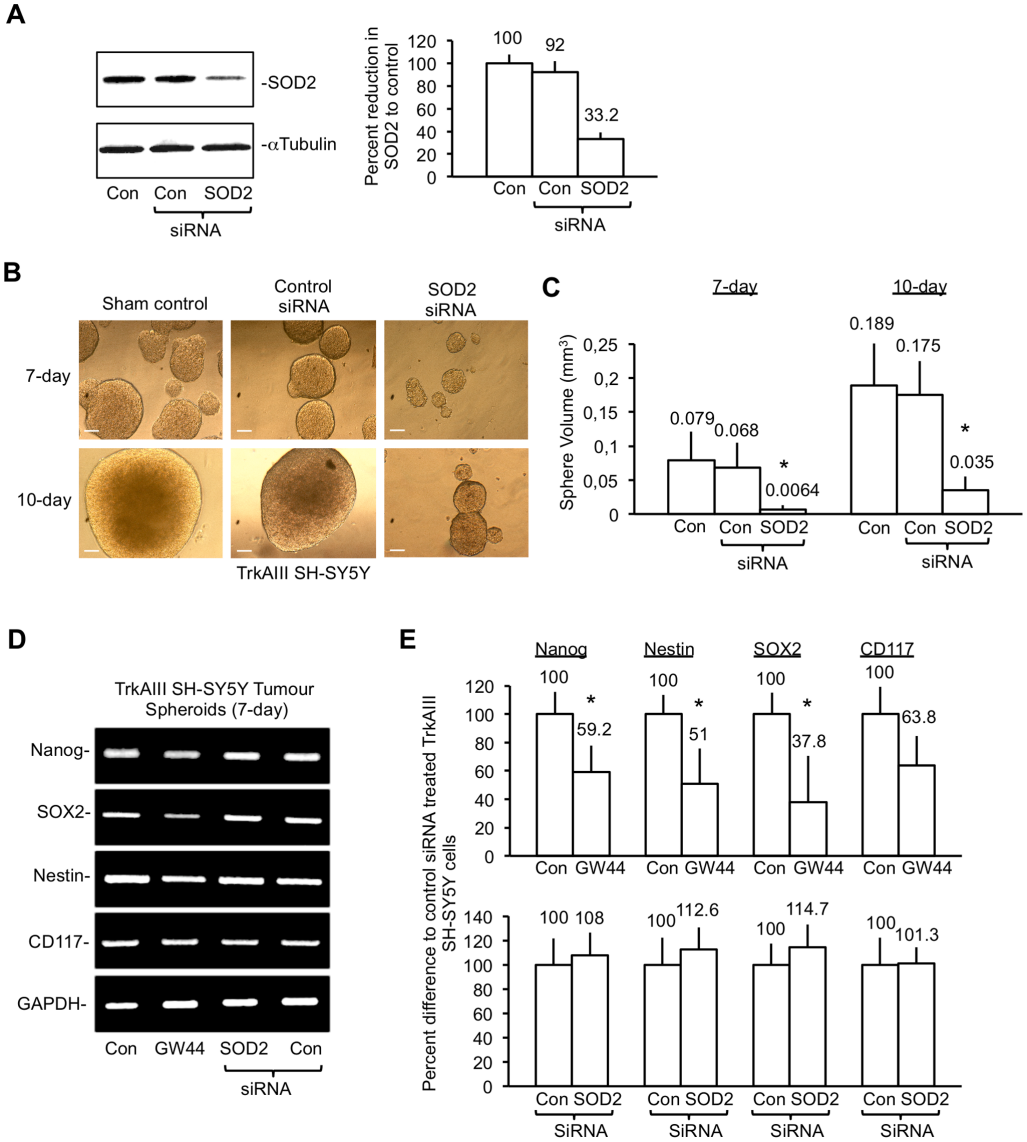
TrkAIII promotion of SH-SY5Y spheroid growth is inhibited by SOD2 knockdown. A) Western blots plus histogram demonstrating SOD2 siRNA knockdown of SOD2 in TrkAIII SH-SY5Y cells, prior to tumour spheroid assay. B) Representative phase contrast micrographs demonstrating the relative sizes of 7-day and 10-day TrkAIII SH-SY5Y tumour spheroids in 48-hour sham transfected controls, 48-hour control siRNA transfected controls and 48-hour SOD2 siRNA transfected TrkAIII SH-SY5Y cells (bars = 100 µm). C) Histogram demonstrating differences in 7-day and 10-day TrkAIII SH-SY5Y tumour spheroid volumes formed by 48-hour sham transfected controls, 48-hour control siRNA transfected controls and 48-hour SOD2 siRNA transfected TrkAIII SH-SY5Y cells. Results are displayed as mean+s.d. volume (mm^3^) of 50 individual tumour spheroids per treatment, in three independent experiments. Mean values are provided above each column and asterisks denote significant differences by Students t-test. D) Representative RT-PCR reactions demonstrating the relative differences in Nanog, SOX2, Nestin, CD117 and GAPDH expressed in 7-day tumour spheroids from untreated control, 1 µM GW441756-treated, 25 nM SOD2 siRNA-treated or 25 nM control siRNA-treated TrkAIII SH-SY5Y cells. E) Histograms demonstrating differences in Nanog, Nestin, SOX2 and CD117 expression in 7-day tumour spheroids from untreated (Con), GW441756-treated (GW44), control siRNA-treated and SOD2 siRNA-treated TrkAIII SH-SY5Y cells. Results are presented as mean±s.d. percent difference in stem cell marker expression, as a densitometric ratio to GAPDH, compared to untreated controls (upper histogram) or control siRNA-treated controls (lower histogram), in three independent experiments. Mean values are presented above each column and asterisks denote significant differences by Student’s t-test.

SiRNA knockdown of SOD2 expression (approximately 70% knockdown) in TrkAIII SH-SY5Y cells (Fig. 9A) significantly reduced the mean volume of 7-day tumour spheroids from 0.079±0.0416 mm^3^ in sham-transfected TrkAIII SH-SY5Y cells and 0.069+0.036 mm^3^ in control siRNA transfected TrkAIII SH-SY5Y cells, to 0.0064±0.007 mm^3^ in SOD2 siRNA treated TrkAIII SH-SY5Y cells (t-test, p = 0.041, df = 4; control siRNA versus SOD2 siRNA treatment) and reduced the mean volume of 10-day tumour spheroids from 0.189±0.09 mm^3^ in sham-transfected TrkAIII SH-SY5Y cells and 0.175±0.08 mm^3^ in control siRNA treated TrkAIII SH-SY5Y cells, to 0.035±0.0175 mm^3^ in SOD2 siRNA treated TrkAIII SH-SY5Y cells (t-test, p = 0.0415, df = 4; control siRNA versus SOD2 siRNA treatment) (Figs. 9B and 9C). Semi quantitative RT-PCR analysis of mRNAs purified from 7-day untreated, 1 µM GW441756-treated, control siRNA and SOD2 siRNA transfected TrkAIII SH-SY5Y tumour spheroids, revealed significant reduction of Nanog (t-test, p = 0.0447, df = 4), Nestin (t-test, p = 0.0403, df = 4); and SOX2 expression (t-test, p = 0.0415, df = 4) and non-significant reduction of CD117 expression in GW441756-treated TrkAIII SH-SY5Y tumour spheroids compared to untreated controls but did not detect significant reduction in either Nanog, Nestin, SOX2 or CD117 expression in SOD2 siRNA treated TrkAIII SH-SY5Y tumour spheroids compared to control siRNA transfected controls (Figs. 9D and 9E).

From these data, we conclude that both TrkAIII tyrosine kinase activity and SOD2 promote the formation of larger SH-SY5Y cell tumour spheroids/spheroid aggregates, in association with increased SOD-2, CD117, Nestin, SOX-2 and Nanog mRNA expression, and that TrkAIII tyrosine kinase activity but not SOD2 promotes Nanog, Nestin and SOX2 neural stem cell marker expression in TrkAIII SH-SY5Y tumour spheroids.

### TrkAIII Promotes Resistance to Rotenone, Paraquat and LY38583-induced Death

Since, TrkAIII promotes SOD2 expression, increases mitochondrial SOD2 activity, augments mitochondrial H_2_0_2_ production, and attenuates induction of mitochondrial free radical ROS accumulation, we compared the susceptibility of pcDNA SH-SY5Y, TrkA SH-SY5Y and TrkAIII SH-SY5Y cells to Rotenone, Paraquat and LY83853-induced ROS-mediated cell death.

**Figure 10:**
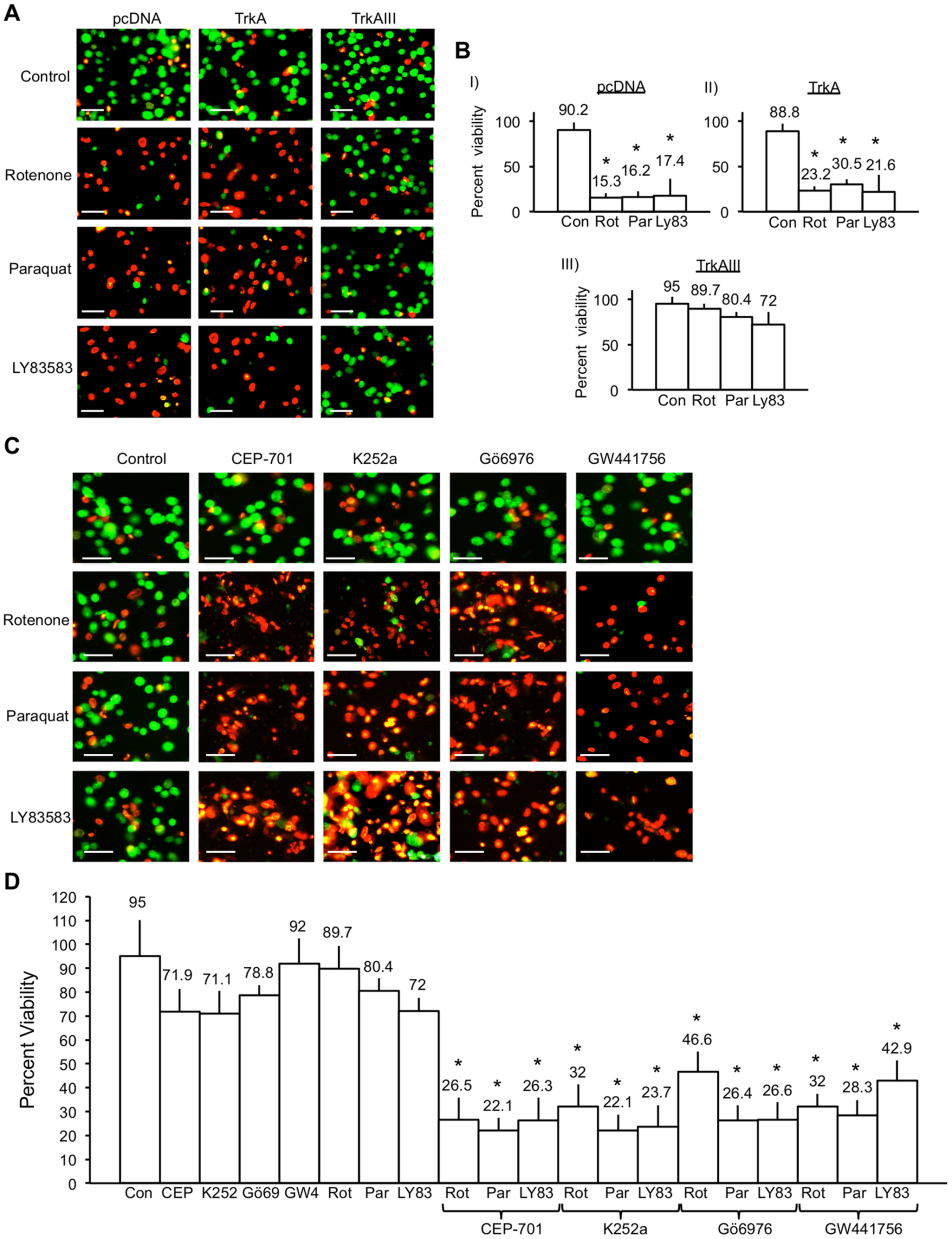
TrkAIII protects SH-SY5Y cells against ROS-mediated death. A) Digital fluorescence micrographs demonstrating changes in viability (green = alive; red = dead) in pcDNA, TrkA and TrkAIII SH-SY5Y cells under basal conditions (Control) or treated for 24 hours at 37°C with either 1 µM Rotenone; 250 µM Paraquat; or for 6 hours with 1 µM LY83583, at 37°C (bar = 100 µm). B) Histograms showing differences in the viability of: (I) pcDNA; (II) TrkAI; and (III) TrkAIII SH-SY5Y cells, treated as described above. Results are displayed as mean±s.d percent viability, in three independent experiments performed in duplicate. Mean values are provided above each column and asterisks denote statistical significance by Student’s t-test comparison of Ros-inducer treated and untreated controls. C) Digital fluorescence micrographs demonstrating viability levels (green = alive; red = dead) in untreated TrkAIII SH-SY5Y cells (Control); TrkAIII SH-SY5Y cells treated with either 1 µM CEP-701, 1 µM K252a, 1 µM Gö6976, 100 nM GW441756, 1 µM Rotenone, 250 µM Paraquat or 1 µM LY83583 alone; or pre-incubated with either 1 µM Cep-701, 1 µM K262a, 1 µM Gö6976 or 100 nM GW441756, then treated with either 1 µM Rotenone, 250 µM Paraquat or 1 µM LY83583 in the continuous presence of each respective inhibitor (Cep-701, K262a, Gö6976 or GW441756), (bar = 100 µm). D) Histogram demonstrating differences in TrkAIII SH-SY5Y cell viability in untreated TrkAIII SH-SY5Y cells (Control); TrkAIII SH-SY5Y cells treated with either 1 µM CEP-701, 1 µM K252a, 1 µM Gö6976, 100 nM GW441756, 1 µM Rotenone, 250 µM Paraquat or 1 µM LY83583 alone; or pre-incubated with either 1 µM Cep-701, 1 µM K262a, 1 µM Gö6976 or 100 nM GW441756, then treated with either 1 µM Rotenone, 250 µM Paraquat or 1 µM LY83583 in the continuous presence of each respective inhibitor, as for Fig. 9C. Results are displayed as mean±s.d percent viability, in three independent experiments performed in duplicate. Mean values are provided above each column and asterisks denote statistical significance by Student’s t-test (p<0.0001, df = 10) and One-Way ANOVA ((2, 15) p<0.0001), comparing inhibitor alone, ROS-inducer alone and inhibitor plus Ros-inducer groups.

PcDNA SH-SY5Y cell basal viability was 90.2±10.6%, TrkA SH-SY5Y cell basal viability was 88.8±12.6% and TrkAIII SH-SY5Y cell basal viability was 95±15.2%, which did not differ significantly (One-way ANOVA: F (2, 15) = 0.379, p = 0.691) (Figs. 10A and 10B).

The viability of TrkAIII SH-SY5Y cells treated 24 hours with 1 µM Rotenone was not significantly reduced from 95±15.2% to 89.7±9.6%, whereas under identical conditions pcDNA SH-SY5Y cells viability was significantly reduced from 90.2±10.6% (control) to 15.3±19.2% (Rotenone) (t-test, p<0.0001, df = 10), and TrkA SH-SY5Y cells viability was significantly reduced from 88.8±12.6% (control) to 23.26±16.5% (Rotenone) (t-test, p<0.0001, df = 10) (One-way ANOVA: F (2, 15) = 40.978, p<0.0001) (Figs. 10A and 10B).

The viability of TrkAIII SH-SY5Y cells treated 24 hours with 250 µM Paraquat was not significantly reduced from 95±15.2% to 80.4±5.3%, whereas under identical conditions pcDNA SH-SY5Y cell viability was significantly reduced from 90.2±10.6% to 16.24±8.3% (t-test, p = 0.0001, df = 10) and TrkA SH-SY5Y cells viability was significantly reduced from 88.8±12.6% to 30.54±7.5% (t Test, p = 0.0001, df = 10) (One way ANOVA: F (2, 15) = 133.27, p<0.0001) (Figs. 10A and 10B).

The viability of TrkAIII SH-SY5Y cells treated 6 hours with 1 µM LY83583 was significantly reduced from 95±15.2% to 72.01±5.5% (t-test, p = 0.0059, df = 10). Under identical conditions pcDNA SH-SY5Y cell viability was significantly reduced from 90.2±10.6% (control) to 17.4±10.8% (LY83583) (t-test, p = 0.0001, df = 10) and TrkA SH-SY5Y cell viability was significantly reduced from 88.8±12.6% (control) to 21.6±9.8% (LY83583) (t-test, p = 0.0001, df = 10). The reduction in TrkAIII SH-SY5Y cell viability induced by LY-83583 was significantly less than that in pcDNA and TrkA SH-SY5Y cells (One-way ANOVA: F (2, 15) = 68.43, p<0.0001) (Figs. 10A and 10B).

As described above, treatment of TrkAIII SH-SY5Y cells with the free radical ROS inducers Rotenone and Paraquat alone did not induce significant TrkAIII SH-SY5Y cell death and LY83583 induced only a subtle but significant increase in TrkAIII SH-SY5Y cell death compared to mock-treated controls. In contrast, Rotenone, Paraquat and LY83583 induced a marked significant increase in TrkAIII SH-SY5Y cell death following pre-treatment with the TrkA tyrosine kinase inhibitors CEP-701, K252a, Gö6976 or GW441756. CEP-701, K252a and Gö6976 alone did not induced more than 25% TrkAIII SH-SY5Y cell death, and GW441756 alone did not induce significant levels of TrkAIII SH-SY5Y cell death (Figs. 10C and 10D).

From these data, we conclude that TrkAIII tyrosine kinase activity is required to inhibit Rotenone, Paraquat or LY83583-induced cell death.

### SiRNA SOD2 Knockdown Re-sensitizes TrkAIII SH-SY5Y Cells to Agents that Induce ROS-Mediated Cell Death

In order to confirm SOD2 involvement in TrkAIII SH-SY5Y cell resistance to agents that induce ROS-mediated cell death, TrkAIII SH-SY5Y cell sensitivity to Rotenone, Paraquat and LY83583 induced death was assessed following siRNA knockdown of SOD2 expression and compared to TrkAIII SH-SY5Y cells treated with control siRNA.

**Figure 11:**
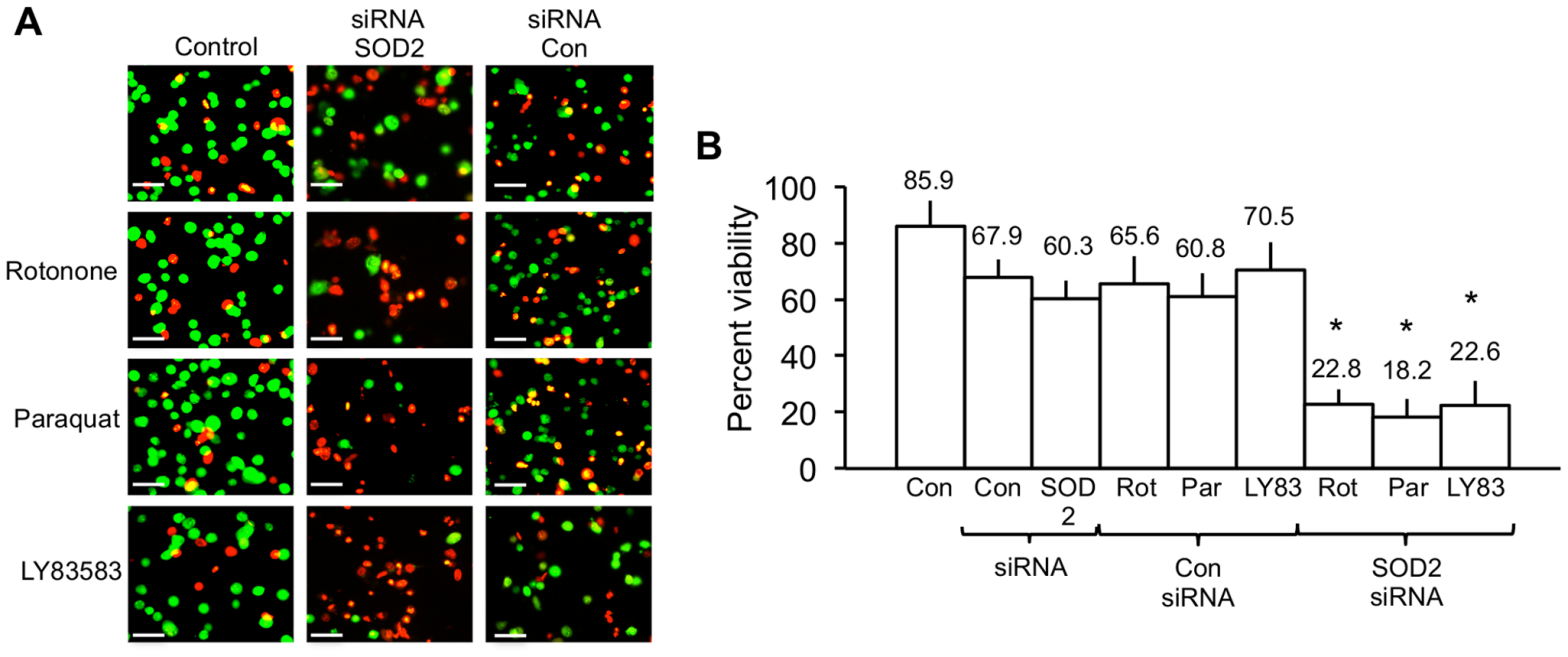
SiRNA SOD2 knockdown restores TrkAIII SH-SY5Y sensitivity to agents that induce ROS-mediated cell death. A) Digital fluorescence micrographs demonstrating cell viability (green = alive; red = dead) in untreated TrkAIII SH-SY5Y cells (control); or TrkAIII SH-SY5Y cells treated for 48 hours with 25 nM control SiRNA or 25 nM SOD2 siRNA alone; or treated for 48 hours with 25 nM control SiRNA or 25 nM SOD2 siRNA then for 24 hours with either 1 µM Rotenone or 250 µM Paraquat, or for 6 hours with 1 µM LY83583 (bar = 100 µm). B) Histogram demonstrating differences in the viability of untreated TrkAIII SH-SY5Y cells (control); TrkAIII SH-SY5Y cells treated for 48 hours with 25 nM control SiRNA or 25 nM SOD2 siRNA alone; or treated for 48 hours with 25 nM control SiRNA or 25 nM SOD2 siRNA then for 24 hours with either 1 µM Rotenone or 250 µM Paraquat, or for 6 hours with 1 µM LY83583. Results are displayed as mean±s.d percent viability, in three independent experiments performed in duplicate. Mean values are provided above each column and asterisks denote statistical significance by Student’s t-test (p<0.0001, df = 10) and One-Way ANOVA ((2, 15) p<0.0001), comparing inhibitor alone, ROS-inducer alone and inhibitor plus Ros-inducer groups.

SiRNA knockdown of SOD2 expression in TrkAIII SH-SY5Y cells restored sensitivity to Rotenone, Paraquat and LY83538-induced death compared to TrkAIII SH-SY5Y cells treated with control siRNA (Figs. 11A and 11B). From these results, we conclude that elevated SOD2 expression in TrkAIII SH-SY5Y cells is largely responsible for attenuating Rotenone, Paraquat, and LY83583-induced death.

## Discussion

In the present study, we report that constitutive expression of the TrkAIII oncogene in human SH-SY5Y NB cells inhibits mitochondrial free radical reactive oxygen species (ROS)-mediated death induced by Rotenone, Paraquat and LY83583, by stimulating SOD2 expression, increasing mitochondrial SOD2 activity and attenuating mitochondrial free radical ROS production. These effects associate with increased mitochondrial capacity to produce H_2_O_2_, occur within the context of a more tumour stem cell-like phenotype, and can be reversed by the specific TrkA tyrosine kinase inhibitor GW441756 [51], by the multi-kinase TrkA inhibitors CEP-701 [59], K252a [49] and Gö6976 [50] and by siRNA knockdown of SOD2 expression, all of which restore the sensitivity of TrkAIII SH-SY5Y cells to Rotenone, Paraquat and LY83583-induced mitochondrial free radical accumulation and free radical-mediated death.

TrkAIII stimulated the expression of SOD2 protein and mRNA in SH-SY5Y cells without altering the expression of antioxidants SOD1, catalase, glutathione peroxidase or thioredoxin-2, indicating relative specificity of action for the antioxidant SOD2. The specific TrkA tyrosine kinase inhibitor GW441756 [51] and the multi-kinase TrkA inhibitors CEP-701, K252a and Gö6976 [49], [50], [59], [70], all of which inhibited TrkAIII activity [this study, 1], inhibited the expression of SOD2 mRNA and protein in TrkAIII SH-SY5Y but not TrkA SH-SY5Y cells. Furthermore, SOD2 expression in SH-SY5Y cells was not stimulated by the expression of Y670/674/675F mutated kinase dead TrkAIII, confirming that stimulation of SOD2 expression was dependent upon TrkAIII tyrosine kinase activity. TrkAIII stimulated the expression of the 4.2 kb relative to 1.5 kb SOD2 mRNA species, within the context of a proliferating, undifferentiated phenotype, suggesting stimulation of SOD2 transcription and supporting a previous report that transcription of the 4.2 kb SOD2 mRNA species predominates in un-differentiated, proliferating cells [34].

SOD2 expression was inhibited by the IP3K inhibitor LY294002 [52], and by the NF-κ B inhibitors PDTC [54] and Bay 11-7082 [55] in TrkAIII SH-SY5Y cells but not in TrkA SH-SY5Y cells, but was not inhibited by the MAPK inhibitor PD098059 [53]. This implicates IP3K and NF-κB but not MAPKs in TrkAIII stimulation of SOD2 expression, supports previous observations that spontaneously active TrkAIII signals through IP3K/Akt/NF-κB but not Ras/MAPK [1] and that SOD2 expression is induced by NF-κB in NB and other cell lines [25], [71] but differs to reports that SOD2 expression is stimulated by the TrkA ligand NGF through rac7, Ki-Ras and CREB transcription factor [14], [15], [47]. Here, we confirm that NGF stimulates SOD2 expression [14], [15], [47] in TrkA SH-SY5Y cells (this study), adding to observations that NGF induces Ras/MAPK signalling and neuronal differentiation in TrkA SH-SY5Y cells [1], and suggesting that SOD2 expression in SH-SY5Y cells could be regulated differently by spontaneously active intracellular TrkAIII and NGF-activated cell surface TrkA receptors. We do not exclude, however, that mechanisms in addition or alternative to NF-κB -mediated SOD2 transcription may regulate SOD2 expression in TrkAIII SH-SY5Y cells, since 4.2 kb SOD2 mRNA is also regulated by small inhibitory RNAs of Alu/7SL origin [35] and by SPB1 binding protein [36]. It is interesting that the IP3K inhibitor LY294002 also inhibited TrkAIII phosphorylation. This was detected following 24 hour but not 3-hour treatments, suggesting an indirect rather than direct inhibitory mechanism and implicating IP3K in the maintenance of constitutive of TrkAIII phosphorylation. We are further investigating this observation.

TrkAIII stimulation of SOD2 expression in SH-SY5Y cells was associated with a more stem cell-like phenotype and not with neuronal differentiation. Previously, SOD2 has been directly implicated in NGF/TrkA-induced neuronal differentiation [14], [15], [47], corroborated by our own observations that NGF stimulates SOD2 expression in TrkA SH-SY5Y cells (this study) in association with neuronal differentiation [1], . This suggests that SOD2 involvement in neuronal differentiation must depend upon additional factors not activated by TrkAIII, which would include Ras/MAPK/Erk signalling induced by NGF-activated TrkA but not spontaneously active TrkAIII in SH-SY5Y cells [1]. In support this, activation of the NGF/TrkA/SOD2/H_2_0_2_ axis within the context of *ki*-Ras/MAPK/Erk signalling has been reported to promote neuronal differentiation by prolonging Erk1/2 transcriptional activity, augmenting Akt activation and inducing cytoskeletal complexes between microtubules, mitochondria and *ki*-Ras containing plasma membranes, in a coordinated mechanism of microtubule assembly and assembly promoting factors [15], [47], [72]. In the absence of Ras/MAPK/Erk signalling, the TrkAIII/SOD2/H_2_0_2_ axis may also regulate the assembly of cytoskeletal components important for maintaining cells in an undifferentiated stem cell-like state. In support of this, TrkAIII interacts with the interphase centrosome and promotes microtubule assembly from centrosomal microtubule organising centre, promoting formation of short peri-nuclear microtubule arrays, similar to those that maintain cells in an undifferentiated state [3], [5], [73].

TrkAIII promotion and maintenance of an undifferentiated SH-SY5Y phenotype [1], [5], is further supported by the increased capacity of TrkAIII SH-SY5Y cells to grow as large tumour spheroids *in vitro,* which is considered to represent cancer stem cell-like behaviour in NB cell lines [66]. The increased size of TrkAIII SH-SY5Y spheroids did not only associate with increased expression of 4.2 kb SOD2 mRNA but also with increased expression of the neural stem cell markers CD117, SOX2, Nestin and Nanog. The possibility that TrkAIII may not only maintain SH-SY5Y cells in an undifferentiated state but may also promote staminality was supported by the reduction of Nanog, Nestin and SOX2 mRNA expression in TrkAIII SH-SY5Y tumour spheroids following incubation with the specific TrkA tyrosine kinase inhibitor GW441756 [51]. This directly implicates TrkAIII in the regulation of SOX2, Nestin and Nanog involvement in NB cancer stem cell biology [74], is consistent with reports that cancer stem cells express higher levels of SOD2 [29], [45], [46] and is, therefore, of relevance to regulation of the cancer stem cell niche in high TrkA expressing unfavourable NBs.

The capacity to grow as tumour spheroids exhibited by control, TrkA and TrkAIII SH-SY5Y cells was completely inhibited by the multi-kinase TrkA inhibitors CEP-701 and K252a [49], [59], [70]. In contrast, the TrkA specific inhibitor GW441756 [51], which inhibited TrkAIII tyrosine phosphorylation and reduced SOD2 expression in TrkAIII SH-SY5Y cells, inhibited spheroid growth exclusively in TrkAIII SH-SY5Y cells reducing spheroid volumes to those formed by TrkA SH-SY5Y cells. These data implicate kinases other than TrkA in SH-SY5Y cell spheroid growth and confirm that TrkAIII promotes the formation of larger SH-SY5Y spheroids. The fact that TrkAIII does not stimulate SH-SY5Y cell proliferation [1], combined with observations of spheroid aggregation by control, TrkA and TrkAIII SH-SY5Y cells, suggests that the formation of larger spheroids by TrkAIII SH-SY5Y cells most likely reflects the increased stress-resistance exhibited by TrkAIII SH-SY5Y cells [this study, 1, 2], rather than increased proliferation and/or spheroid-aggregation. Within the context of large tumour spheroid-aggregates, stress would result from reduced oxygen and nutrient diffusion [75], which would be expected to regulate spheroid size on the basis of relative resistance to stress. SiRNA knockdown of SOD2 expression significantly reduced mean TrkAIII SH-SY5Y tumour spheroid volume but, unlike GW441756, did not reduce Nanog, Nestin, SOX2 or CD117 expression in tumor spheroids, implicating SOD2 in the promotion of SH-SY5Y tumour spheroid growth and survival but not staminality. The TrkAIII/SOD2 axis may, therefore, act in an analogous but opposite way to the NGF/TrkA/SOD2 axis, by driving NB staminality and survival rather than neuronal differentiation and survival [1], [4], [12]–[15], [47], [76].

The increased levels of mitochondrial SOD2 protein and activity in TrkAIII SH-SY5Y cells is consistent with the mitochondrial translocation sequence present within SOD2 and confirms that TrkAIII does not inhibit SOD2 activity, which is negatively regulated by tyrosine nitration, serine/threonine phosphorylation and lysine acetylation [17], [18], [37]. Consistent with high mitochondrial SOD2 levels, TrkAIII SH-SY5Y cells exhibited attenuation of mitochondrial free radical ROS accumulation induced by the mitochondrial free radical ROS inducing agents Rotenone [56], Paraquat [57] or LY83583 [58]. Furthermore, highly purified mitochondria from TrkAIII SH-SY5Y cells also exhibited increased capacity to produce H_2_0_2_ in response to succinate, also consistent with increased mitochondrial SOD2 activity. The sensitivity of TrkAIII SH-SY5Y cells to Rotenone, Paraquat and LY83583-induced mitochondrial ROS accumulation could be restored by the specific TrkA inhibitor GW441756, the multi-kinase TrkA inhibitors CEP-701, K252a and Gö6976, and also by siRNA knockdown of SOD2 expression, confirming that resistance to Rotenone, Paraquat and LY83583-induced mitochondrial ROS accumulation was TrkAIII tyrosine kinase-mediated and SOD2 dependent.

Since the unbridled accumulation of both free radical and non-free radical ROS causes oxidative damage and cellular death [16], [21], [77], [78], we compared the relative sensitivity of pcDNA, TrkA and TrkAIII SH-SY5Y cells to Rotenone, LY83583 and Paraquat-induced ROS-induced death. TrkAIII SH-SY5Y cells were significantly more resistant to Rotenone, LY83583 and Paraquat-induced death than either pcDNA SH-SY5Y or TrkA SH-SY5Y cells, indicating that mitochondrial free radical ROS rather than non-free radical H_2_0_2_ were largely responsible for cell death under these conditions. The sensitivity of TrkAIII SH-SY5Y cells to Rotenone, Paraquat and LY83583-induced death was also restored by the specific TrkA inhibitor GW441756, the muti-kinase TrkA inhibitors CEP-701, K252a and Gö6976 and by siRNA knockdown of SOD2 expression. These observations extend our previous report that TrkAIII protects SH-SY5Y cells against doxorubicin-induced death [1], also mediated by mitochondrial free radical ROS [22], and confirm that TrkAIII protection of SH-SY5Y cells against Rotenone, LY83583 and Paraquat-induced death results from the attenuation of mitochondrial free radical ROS accumulation, is TrkAIII tyrosine kinase-mediated and SOD2 dependent.

The increased capacity of mitochondria from TrkAIII SH-SY5Y cells to produce H_2_0_2_, indicates that SH-SY5Y cells have an adequate capacity to detoxify H_2_0_2_, which is supported by the high levels of the H_2_0_2_ detoxifiers catalase and glutathione peroxidase expressed by SH-SY5Y cells. Mitochondrial H_2_0_2_ has been implicated in malignant progression, augments genetic instability by increasing sister chromatid exchange and chromosomal translocation [79], [80], increases MMP-expression and MMP-dependent invasion and promotes metastatic dissemination [28], [40]–[42]. TrkAIII also augments genetic instability by increasing sister chromatid exchange and chromosomal instability, increases MMP-9 expression and promotes metastatic growth [1]–[4]. We are currently investigating whether these oncogenic effects of TrkAIII are also mediated by an increase in mitochondrial SOD2-mediated H_2_0_2_ production.

In conclusion, our study shows that TrkAIII promotes SH-SY5Y NB cell resistance to agents that induce mitochondrial free radical ROS-mediated death by up-regulating SOD2 expression, increasing mitochondrial SOD2 activity and attenuating the accumulation of mitochondrial free radical ROS, in association with up-regulated mitochondrial capacity to produce H_2_0_2_ and within the context of a more tumour stem cell-like phenotype. This novel TrkAIII/SOD2 axis function suggest that the combined use of TrkAIII and/or SOD2 inhibitors, together with radiotherapy or chemotherapeutic agents that induce mitochondrial free radical ROS-mediated death, could provide a therapeutic advantage that may not only enhance tumour cytotoxicity but may also reduce malignant behaviour and target the stem cell niche in high TrkA expressing unfavourable NBs.
